# Prospective association between biological aging and risk of hospital-diagnosed MASLD: evidence from the UK Biobank

**DOI:** 10.3389/fendo.2026.1853319

**Published:** 2026-06-17

**Authors:** Xue Yang, Sicheng Li, Qingping Xue, Peijing Yan, Qian Li, Yi Gong, Xiayue Fan, Wenzhi Zhu, Shiyi Wu, Shanshan Zhang, Ko Willems van Dijk, Patrick C.N. Rensen, Ruifang Li-Gao, Yanan Wang, Ting Yao

**Affiliations:** 1 Department of Endocrinology, The First Affiliated Hospital of Xi’an Jiaotong University, Xi’an, Shaanxi, China; 2 Med-X Institute, Center for Immunological and Metabolic Diseases, the First Affiliated Hospital of Xi’an Jiaotong University, Xi’an, Shaanxi, China; 3 Xiamen Cardiovascular Hospital of Xiamen University, School of Medicine, Xiamen University, Xiamen, Fujian, China; 4 Department of Epidemiology and Biostatistics, School of Public Health, Chengdu Medical College, Chengdu, Sichuan, China; 5 Clinical Research Center, Sichuan Provincial People’s Hospital, University of Electronic Science and Technology of China, Chengdu, Sichuan, China; 6 Department of Healthcare-associated Infection Management, Chengdu Hi-Tech Zone Hospital for Women and Children (Chengdu Hi-Tech Zone Hospital for Maternal and Child Healthcare), Chengdu, Sichuan, China; 7 Department of Epidemiology and Biostatistics, West China School of Public Health and West China Fourth Hospital, Sichuan University, Chengdu, Sichuan, China; 8 Department of Human Genetics, Leiden University Medical Center, Leiden, Netherlands; 9 Department of Medicine, Division of Endocrinology, and Einthoven Laboratory for Experimental Vascular Medicine, Leiden University Medical Center, Leiden, Netherlands; 10 Department of Clinical Epidemiology, C7-P, Leiden University Medical Center, Leiden, Netherlands; 11 Department of Physiology and Pathophysiology, School of Basic Medical Sciences, Xi’an Jiaotong University Health Science Center, Xi’an, Shaanxi, China

**Keywords:** age acceleration, biological aging, lifestyle, MASLD, mediation

## Abstract

**Background and aims:**

Metabolic dysfunction-associated steatotic liver disease (MASLD) is a prevalent metabolic disorder linked to increased all-cause and cardiovascular mortality. While accelerated biological aging is a known risk factor for age-related diseases, its role in MASLD remains unclear. This study explores the association between biological aging and hospital-diagnosed MASLD and investigates the potential mediating effects of biological aging on lifestyle-MASLD relationships.

**Methods and results:**

Data were from the UK Biobank, and the biological age was estimated by PhenoAge and Klemera-Doubal method age (KDMAge). The association between biological aging and hospital-diagnosed MASLD (defined as hospital admission or death) was estimated using Cox regression. Biological aging acceleration was defined as positive residuals obtained from regressing biological age on chronological age. Mediation analyses were used to assess the potential mediating role of biological aging in the relationships between lifestyle and hospital-diagnosed MASLD. Among 247,444 participants, 3,254 developed hospital-diagnosed MASLD during a median follow-up of 13.7 years. Accelerated biological aging was significantly associated with hospital-diagnosed MASLD with hazard ratios of 1.46 (95% confidence interval, 1.35, 1.57) for PhenoAge acceleration and 1.35 (1.19, 1.53) for KDMAge acceleration. In mediation analyses, PhenoAge acceleration significantly accounted for the associations between four unhealthy lifestyle factors (smoking, drinking, poor diet, and low physical activity) and MASLD, with mediation proportions ranging from 11.4% to 25.5%, and the strongest effect observed for smoking. In contrast, KDMAge acceleration showed minimal mediation effects (≤2%).

**Conclusions:**

Accelerated biological aging was associated with hospital-diagnosed MASLD and may partially mediate the associations between unhealthy lifestyles and hospital-diagnosed MASLD. These findings support the potential relevance of biological aging in MASLD risk stratification and prevention.

## Introduction

Metabolic dysfunction-associated steatotic liver disease (MASLD) has emerged as a prevalent and concerning health issue globally, defined by the presence of hepatic steatosis together with cardiometabolic risk factors (1). The global prevalence of MASLD is around 25% of the adult population, and its incidence has shown a notable rise (2). These estimates are largely derived from non-alcoholic fatty liver disease (NAFLD) studies, as previous evidence suggests that the differences between NAFLD and MASLD are minimal and findings remain largely applicable under the updated definition (3). MASLD encompasses a spectrum of liver conditions, ranging from simple steatosis to non-alcoholic steatohepatitis, which may progress to advanced fibrosis, cirrhosis, and hepatocellular carcinoma, thereby posing substantial burdens on healthcare systems and public health worldwide (2, 4, 5). The etiology of MASLD is multifactorial, involving intricate interplays between genetic predisposition, environmental factors, and various metabolic disturbances (6). Among these, aging has garnered increasing attention as a crucial risk factor for MASLD (7, 8). Aging is associated with various physiological changes, including alterations in lipid metabolism, insulin resistance, and chronic low-grade inflammation, which closely mirror the metabolic dysregulations observed in MASLD (4, 9, 10). Hence, clarifying the association between biological age and MASLD is important for understanding the pathogenesis of MASLD.

Aging is a multifactorial biological process characterized by the progressive decline in the structural integrity and functional robustness of cells, tissues, and organs (11). Hence, chronological age alone is insufficient to reflect the state of biological systems (11). Several biological age clocks, such as telomere length, epigenetic aging, and phenotypic age, have been developed to reflect biological age. Among these, algorithms integrating data from clinical parameters have demonstrated the highest accuracy in predicting morbidity and mortality (12–14). PhenoAge and Klemera-Doubal method age (KDMAge) are two published and widely validated algorithms for biological age for the prediction of mortality (15) and chronic diseases, such as rheumatoid arthritis, chronic respiratory diseases, depression, and anxiety (14, 16, 17). Several age-related phenotypes, such as telomere length (18) and DNA methylation age (19), showed significant associations with MASLD risk; however, the associations of PhenoAge and KDMAge with MASLD are still unclear.

Biological aging is closely related to physical functions and is susceptible to external factors, for which it acts as an important mediator in the associations between environmental factors and diseases (20, 21). Smoking, drinking, diet, and physical activity are established influential factors for MASLD (22). Previous studies also reported close relationships between lifestyle factors and accelerated aging (23–25). Exploring the potential mediating role of biological aging in the relationship between lifestyle factors and MASLD offers valuable insights for health management and disease prevention. However, comprehensive studies on this topic remain scarce.

Therefore, this study had three objectives. First, we investigated the prospective association between biological aging and hospital-diagnosed MASLD. Second, we examined whether these associations were consistent across different biological aging measures, including PhenoAge and KDMAge. Third, we further assessed the potential mediating role of biological aging in the relationships between unhealthy lifestyles and the risk of hospital-diagnosed MASLD.

## Methods

### Study populations

This study enrolled participants in the UK Biobank study. The UK Biobank is designed as a large prospective study with over 500,000 participants aged 37–73 years. The baseline survey was conducted between 2006 and 2010, including a touch-screen questionnaire (i.e., demographic characteristics, lifestyle, behaviors, medical condition, etc.), anthropometry, and biological sampling. The details of study designs have been described elsewhere (http://www.ukbiobank.ac.uk).

In this study, we conducted prospective analyses and included all participants with available data on biological aging and MASLD. In our prospective analyses of incident hospital-diagnosed MASLD, we excluded participants who withdraw consent (n = 191), pregnant women (n = 150), participants with other prevalent liver disease (n = 1,819), those missed data on PhenoAge or KDMAge calculation (n = 176,716), participants with missing or unknown sociodemographic information, such as race and ethnicity (n = 1,475), education (n = 3,224), income (n = 373), body mass index (BMI; n = 639), smoking status (n = 1,047), alcohol consumption (n = 271), and physical activity (n = 68,630). In addition, we also excluded people with prevalent MASLD at the baseline survey (n = 185). Given the reliance on hospital data, this definition likely captures only clinically diagnosed cases and may not include individuals with undiagnosed or subclinical MASLD. Finally, 247,444 participants were included in the primary analyses, and the flow chart of participant selection is shown in **Supplementary Figure 1**.

The UK Biobank study was conducted following both the Declarations of Helsinki and Istanbul and approved by the North West Multi-Centre Research Ethics Committee. All participants in the UK Biobank provided written informed consent. The data required in this study were obtained by an application to the UK Biobank (application number 185057).

### Assessment of biological aging

We assessed biological age using two widely validated algorithms applicable to the data available in the UK Biobank, namely PhenoAge and KDMAge. PhenoAge was calculated according to the algorithm previously developed by Levine et al. (15), based on chronological age and nine clinical biomarkers, including concentrations of albumin, creatinine, glucose, C-reactive protein (CRP), alkaline phosphatase, lymphocyte percentage, mean cell volume, red cell distribution width (RDW), and white blood cell count. KDMAge was calculated using the Klemera–Doubal method based on chronological age and multiple physiological parameters, including forced expiratory volume in one second (FEV1), systolic blood pressure, and seven blood chemistry biomarkers (albumin, alkaline phosphatase, blood urea nitrogen, creatinine, CRP, glycated hemoglobin [HbA1c], and total cholesterol). PhenoAge and KDMAge were computed using the R package “BioAge”, which implements previously published and validated algorithms (**Supplementary Methods**). Details of the variables, UK Biobank field IDs, units, and unit conversions used for the calculation of PhenoAge and KDMAge are provided in **Supplementary Table 1**.

To qualify biological aging independent of chronological age, we regressed biological age on chronological age, and the residuals from these models were used to represent biological age acceleration (PhenoAge acceleration and KDMAge acceleration). Accelerated aging was defined as a residual value of 0 or greater. This residual-based approach has been widely used in previous studies of biological aging. Biological age acceleration values were standardized using a z-score transformation (mean = 0, standard deviation = 1) before continuous analyses. Hazard ratios (HRs) were therefore estimated per one standard deviation (SD) increase in biological age acceleration, providing a scale-free effect estimate that facilitates interpretation and comparison of associations across models.

### Ascertainment of hospital-diagnosed MASLD

The term MASLD replaced NAFLD in 2023 (1). Previous studies have estimated that approximately 99% of individuals previously classified as having NAFLD would meet the MASLD criteria, despite differences in definition (26). Thus, in our study, we defined hospital-diagnosed MASLD as a hospital admission or death cause with ICD-10 codes (International Classification of Diseases, 10th revision) of K76.0 (fatty (change of) liver, not elsewhere classified) and K75.8 (other specified inflammatory liver diseases) at baseline survey and during follow-up (**Supplementary Table 2**), consistent with the ICD-based definitions previously used for NAFLD in UK Biobank studies. This case ascertainment approach primarily captures individuals with MASLD severe enough to result in hospital contact and clinical coding, and therefore reflects clinically recognized cases. To improve specificity, we excluded participants with prevalent liver diseases that may represent alternative etiologies, including viral or autoimmune hepatitis, biliary cirrhosis, alcohol-related liver disease, and other chronic liver conditions, based on hospital records and self-reported data (**Supplementary Table 2**). Hospital admission data were available until October 2022. Therefore, the follow-up was censored on this date or the date of death, which occurred earlier.

### Measurement of cardiometabolic biomarkers

Cardiometabolic biomarkers included five aspects: glucose homeostasis, lipid profile, blood pressure, inflammation, and liver function. For glucose homeostasis, blood glucose was measured by hexokinase analysis on a Beckman Coulter AU5800. HbA1c was tested through HPLC analysis on a Bio-Rad VARIANT II Turbo. Triglyceride-glucose (TyG) index was determined as ln [triglyceride (mg/dL) × glucose (mg/dL)/2]. For lipid profiles, triglyceride (TG) and total cholesterol (TC) were measured by CHO-POD analysis on a Beckman Coulter AU5800. High-density lipoprotein cholesterol (HDL-C) was measured by enzyme immunoinhibition analysis on a Beckman Coulter AU5800, while low-density lipoprotein cholesterol (LDL-C) was measured by enzymatic protective selection analysis on a Beckman Coulter AU5800. Blood pressure was measured using an Omron device and two measures of blood pressure were taken a few moments apart. Gamma-glutamyl transferase (GGT), CRP, and serum urate were inflammatory indicators. GGT was tested by either a Hitachi Model 917 multichannel analyzer (Roche Diagnostics, Indianapolis, IN, USA) or LX20 chemistry analyzer (Beckman Coulter, Brea, CA, USA). CRP was analyzed using immunoturbidimetric-high sensitivity analysis on a Beckman Coulter AU5800. Urate was measured by uricase PAP analysis on a Beckman Coulter AU5800. Aspartate aminotransferase (AST) and alanine aminotransferase (ALT) were measured by IFCC analysis on a Beckman Coulter AU5800.

### Measurement of covariates

Sociodemographic characteristics and lifestyles, such as age (year, continuous), sex (male and female), race/ethnicity (White and others), education (less than high school, high school or equivalent, and college and above), Townsend deprivation index (continuous), BMI (kg/m^2^, continuous), smoking status (never, previous, and current), alcohol consumption (never, previous, and current), physical activity (low, moderate, and high), and diet quality, were determined as covariates. Physical activity was evaluated by the International Physical Activity Questionnaire. The diet quality was assessed through a dietary recommendation for cardiovascular health. Participants meeting at least five of the recommendations were classified as having a healthy diet (27), whereas those meeting fewer than five recommendations were considered to have a poor diet. Hypertension was ascertained by self-report/ICD 10-diagnosis and/or blood pressure ≥ 140/90 mm Hg. Diabetes was determined by self-report/ICD 10-diagnosis and/or blood glucose ≥ 11.1 mmol/L and/or HbA1c ≥ 48 mmol/mol. Dyslipidemia was determined by self-report/ICD 10 diagnosis and/or non-HDL cholesterol ≥ 160 mg/dL (28).

### Statistical analyses

Continuous variables were summarized as mean (SD), and categorical variables were presented as numbers and percentages (n, %). The student’s t-test and chi-square test were used to compare continuous and categorical characteristics between participants with and without incident hospital-diagnosed MASLD, respectively. Comparisons of characteristics across participants with and without accelerated aging were also performed.

We used multivariable Cox proportional hazard regression to estimate the HRs and their 95% confidence intervals (CIs) for the associations between biological aging and incident hospital-diagnosed MASLD. The proportional hazards assumption was evaluated using Schoenfeld residual methods, and the results suggested no violations (e.g., *P* = 0.103 and 0.139 for PhenoAge acceleration and KDMAge acceleration, respectively). The PhenoAge acceleration and KDMAge acceleration were modeled as continuous (per SD increase) and categorical (by tertiles and accelerated aging) variables, respectively. We calculated the cumulative incidence of hospital-diagnosed MASLD across different levels of PhenoAge acceleration and KDMAge acceleration. The non-linear associations were estimated using the restricted cubic splines with four knots located at the 5th, 35th, 65th, and 95th percentiles. HRs and corresponding 95% CIs were estimated using the median value as the reference. The *P* values for non-linearity were calculated to assess departures from linear associations. Three multivariable Cox proportional hazards regression models were fitted. Covariates were selected *a priori* based on previous literature and their potential roles as confounders in the association between biological aging and hospital-diagnosed MASLD, and were adjusted in a stepwise manner. In Model 1, we adjusted for age, sex, race, education, and income. In Model 2, we further adjusted for BMI, smoking status, alcohol consumption, physical activity, healthy diet, and covariates adjusted in Model 1. Because hypertension, diabetes, and dyslipidemia may partially lie along the pathway linking biological aging and MASLD, they were additionally adjusted for in sensitivity analyses to evaluate whether the observed associations were independent of major metabolic comorbidities (**Supplementary Figure 2**). In addition to primary analyses of MASLD risk, we conducted secondary analyses using linear regression models to evaluate the associations between biological aging and cardiometabolic biomarkers, in order to further characterize the metabolic profiles associated with accelerated biological aging.

Exploratory subgroup analyses were conducted across age (< 60 and ≥ 60 years), sex (male and female), obesity status (normal, overweight, and obesity), physical activity (low, moderate, and heavy), and healthy diet (yes and no). Stratified factors were grouped according to established clinical criteria. The interaction effects of biological aging with stratified factors were evaluated by comparing models with and without interaction terms and then tested using the Likelihood Ratio test.

We performed several other sensitivity analyses to examine the robustness of associations observed in the primary analyses. First, we assessed the cross-sectional associations between biological aging and alternative MASLD definitions, including imaging-defined MASLD based on proton density fat fraction (PDFF) ≥5% and fatty liver index (FLI)-defined MASLD based on FLI ≥60 together with at least 1 cardiometabolic risk factor and low alcohol consumption (<20 [female]/30 [male] g/day), according to a previously published UK Biobank study (29). Second, we excluded participants who developed hospital-diagnosed MASLD within the first 5 years of follow-up. Third, we excluded participants who reported excessive alcohol consumption, which was defined as a weekly consumption exceeding 14 units for men and women (30). Fourth, to evaluate the potential impact of missing covariates, multiple imputation by chained equations was performed under the missing at random assumption using the R package “mice”. Five imputed datasets with 10 iterations were generated using variable type–specific methods (predictive mean matching for continuous variables, logistic regression for binary variables, and polytomous regression for categorical variables). All variables included in the analyses, including exposure, outcome, follow-up time, and covariates, were incorporated into the imputation models as predictors. Variables without missing data were retained as predictors but were not imputed. Post-imputation diagnostics showed that the distributions of imputed variables were comparable to those of the observed data. Cox regression analyses were repeated across the imputed datasets, and pooled estimates were combined using Rubin’s rules.

The predictive ability of biological aging for hospital-diagnosed MASLD was assessed by the area under the receiver operating characteristic curve (AUROC) of logistic regression models. First, we evaluated the predictive ability of biological age acceleration and chronological age alone for MASLD risk. Then, we further evaluated the additive values of including biological age acceleration and chronological age into the base model, which was established using BMI, AST, and ALT. Furthermore, Delong test was conducted to compare the predictive performance of different models.

The associations between smoking, drinking, diet, or physical activity and hospital-diagnosed MASLD were estimated by multivariable Cox proportional hazard regression, while logistic regression models were used to evaluate the relationships between these lifestyles and biological age acceleration. Before mediation analyses, the proportional hazards assumption was assessed using Schoenfeld residual diagnostics, and multicollinearity among covariates was evaluated using variance inflation factors (VIFs), which indicated negligible collinearity (all VIFs ranged from 1.007 to 1.033). Mediation analyses were conducted under the assumption of no unmeasured confounding after adjustment for measured covariates. Mediation analyses were performed using the R package “CMAverse” with a regression-based approach (model = “rb”) to investigate the potential mediating role of biological age acceleration in the associations between lifestyle factors and hospital-diagnosed MASLD. Logistic regression was specified for the mediator model and Cox proportional hazards regression for the outcome model. Models were adjusted for age, sex, race and ethnicity, education, Townsend deprivation index, BMI, current smoking status, alcohol consumption, physical activity, and healthy diet, as appropriate for each exposure–mediator–outcome pathway. Under the counterfactual framework, the total effect was decomposed into natural direct and indirect effects, and the mediation proportion was estimated to quantify the proportion of the lifestyle–MASLD association explained by biological aging. Effect estimates and 95% CIs were obtained using the parametric functional approach with delta-method inference (estimation = “paramfunc”; inference = “delta”). Mediation analyses were conducted using complete-case data, and CMAverse’s internal multiple-imputation procedure was not used (multimp = FALSE). Lifestyle factors were assessed at baseline, biological aging acceleration was treated as a mediator, and incident MASLD was subsequently ascertained during follow-up. Given the large sample size of the present study, the mediation analyses were considered to have adequate statistical power to detect modest indirect effects. As lifestyle factors, biological aging, and covariates were measured at the same baseline visit, these analyses were considered exploratory in nature and should not be interpreted as evidence of causal mediation.

Data analyses were done using R 4.2.3. Two-tailed *P* values less than 0.05 were considered statistically significant.

## Results

### Basic characteristics

Baseline characteristics of the study participants were reported in **Table 1**. In detail, among 247,444 participants included, the mean age was 56.08 (standard deviation: 8.12), and 119,423 were male (48.3%). Over a median follow-up of 13.7 years, 3,254 participants developed hospital-diagnosed MASLD. Participants with incident hospital-diagnosed MASLD were older, more likely to be male, other race, and previous/current smokers, or to have lower income, less physical activity, poor diet, hypertension, and diabetes (all *P* values ≤ 0.005). People with hospital-diagnosed MASLD also showed higher biological age and age acceleration (all *P* values < 0.001; **Table 1**; **Supplementary Figure 3**). A comparison of the basic characteristics across PhenoAge acceleration is shown in **Supplementary Table 3**. The baseline characteristics of participants included and excluded from the final analyses are presented in **Supplementary Table 4**. Although several characteristics differed statistically between the two groups, the overall distributions were broadly comparable. The baseline characteristics of participants with and without PDFF measurements are shown in **Supplementary Table 5**.

**Table 1:** Baseline characteristics of participants from UK Biobank (N = 247,444).^*^

Characteristics	Total (N = 247,444)	No hospital-diagnosed MASLD (N = 244,190)	Incident hospital-diagnosed MASLD (N = 3,254)	*P* value
Chronological age, years	56.08 (8.12)	56.07 (8.12)	56.63 (7.94)	<0.001
Sex				<0.001
Male	119,423 (48.3%)	117,735 (48.2%)	1,688 (51.9%)	
Female	128,021 (51.7%)	126,455 (51.8%)	1,566 (48.1%)	
Race and ethnicity				0.005
White ethnicity or race	236,288 (95.5%)	233,214 (95.5%)	3,074 (94.5%)	
Others	11,156 (4.5%)	10,976 (4.5%)	180 (5.5%)	
Education				<0.001
Less than high school	32,861 (13.3%)	32,188 (13.2%)	673 (20.7%)	
High school or equivalent	125,371 (50.7%)	123,598 (50.6%)	1,773 (54.5%)	
College and above	89,212 (36.1%)	88,404 (36.2%)	808 (24.8%)	
Townsend deprivation index	-1.51 (2.96)	-0.58 (3.39)	-1.50 (2.97)	<0.001
Body mass index, kg/m^2^	27.18 (4.58)	27.13 (4.55)	31.98 (5.25)	<0.001
Obesity status				<0.001
Normal	85,137 (34.4%)	84,817 (34.7%)	320 (9.8%)	
Overweight	106,686 (43.1%)	105,442 (43.2%)	1,244 (38.2%)	
Obesity	55,621 (22.5%)	53,931 (22.1%)	1,690 (51.9%)	
Smoking status				<0.001
Never	136,971 (55.4%)	135,514 (55.5%)	1,457 (44.8%)	
Previous	86,479 (34.9%)	85,151 (34.9%)	1,328 (40.8%)	
Current	23,994 (9.7%)	23,525 (9.6%)	469 (14.4%)	
Alcohol consumption				<0.001
Never	9,045 (3.7%)	8,883 (3.6%)	162 (5.0%)	
Previous	7,736 (3.1%)	7,550 (3.1%)	186 (5.7%)	
Current	230,663 (93.2%)	227,757 (93.3%)	2,906 (89.3%)	
Physical activity				<0.001
Low	45,023 (18.2%)	44,234 (18.1%)	789 (24.2%)	
Moderate	100,649 (40.7%)	99,366 (40.7%)	1,283 (39.4%)	
High	101,772 (41.1%)	100,590 (41.2%)	1,182 (36.3%)	
Healthy diet, yes	37,758 (15.3%)	37,376 (15.3%)	382 (11.7%)	<0.001
Hypertension, yes	121,271 (49.0%)	119,272 (48.8%)	1,999 (61.4%)	<0.001
Diabetes, yes	10,285 (4.2%)	9,818 (4.0%)	467 (14.4%)	<0.001
Dyslipidemia, yes	132,767 (53.7%)	130,974 (53.6%)	1,793 (55.1%)	0.100
Biological age				
PhenoAge, years	44.96 (10.04)	44.92 (10.04)	48.27 (10.09)	<0.001
PhenoAge residual	-0.17 (5.39)	-0.20 (5.37)	2.56 (6.05)	<0.001
KDMAge, years	59.60 (8.42)	59.57 (8.42)	61.36 (8.37)	<0.001
KDMAge residual	0.02 (3.93)	-0.001 (3.92)	1.28 (4.63)	<0.001
PhenoAge acceleration				<0.001
PhenoAge, non-accelerated aging	137,927 (55.7%)	136,775 (56.0%)	1,152 (35.4%)	
PhenoAge, accelerated aging	109,517 (44.3%)	107,415 (44.0%)	2,102 (64.6%)	
KDMAge acceleration				<0.001
KDMAge, non-accelerated aging	164,687 (66.6%)	162,821 (66.7%)	1,866 (57.3%)	
KDMAge, accelerated aging	82,757 (33.4%)	81,369 (33.3%)	1,388 (42.7%)	
Components of biological ages				
FEV1, L	2.79 (0.79)	2.79 (0.79)	2.64 (0.77)	<0.001
SBP, mm Hg	137.20 (18.38)	137.19 (18.38)	140.46 (18.05)	<0.001
Total cholesterol, mg/dL	220.48 (43.52)	220.59 (43.46)	212.41 (47.55)	<0.001
HbA1c, %	5.42 (0.58)	5.42 (0.57)	5.72 (0.89)	<0.001
Blood urea nitrogen, mg/dL	15.09 (3.76)	15.08 (3.76)	15.20 (4.05)	0.109
Lymphocyte, %	29.00 (7.38)	29.00 (7.38)	28.91 (7.41)	0.500
Mean cell volume, fL	82.79 (5.28)	82.79 (5.27)	82.48 (5.74)	0.002
Serum glucose, mmol/L	5.09 (1.16)	5.09 (1.15)	5.58 (1.96)	<0.001
Red cell distribution width, %	13.45 (0.94)	13.45 (0.94)	13.56 (1.03)	<0.001
White cell count, 1000 cells/μL	6.82 (1.89)	6.81 (1.89)	7.36 (1.87)	<0.001
Albumin, g/L	4.53 (0.26)	4.53 (0.26)	4.51 (0.28)	<0.001
Creatinine, mg/dL	0.82 (0.18)	0.82 (0.18)	0.83 (0.21)	0.067
C-reactive protein, mg/dL	0.24 (0.39)	0.24 (0.39)	0.38 (0.49)	<0.001
Alkaline phosphatase, U/L	82.18 (25.08)	82.05 (24.86)	92.16 (36.50)	<0.001

FEV1, forced expiratory volume in 1-second; HbA1c, glycosylated hemoglobin; KDM, Klemera-Doubal method; MASLD, metabolic dysfunction-associated steatotic liver disease; SBP, systolic blood pressure.

^*^ Continuous variables were presented as mean (standard deviation) and categorical variables were presented as number (percent, %). *P* values were calculated using the Student’s *t*-test and chi-square test for continuous and categorical characteristics, respectively.

### Associations between biological aging and incident hospital-diagnosed MASLD

**Figure 1:**
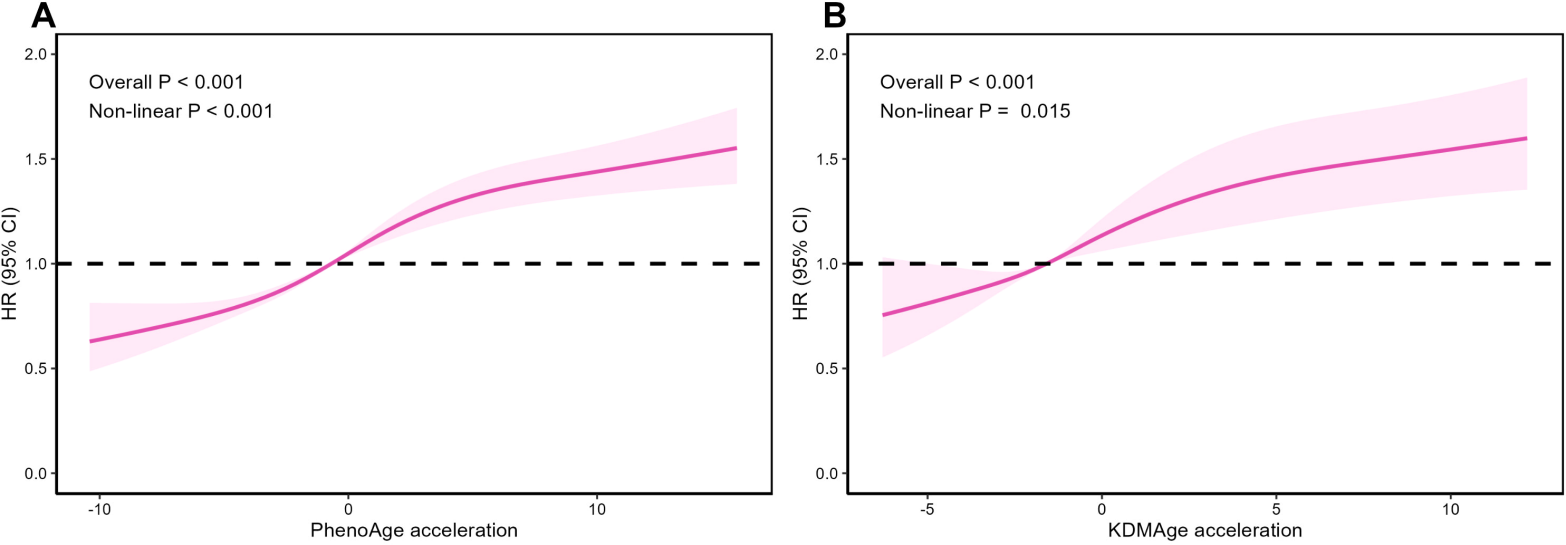
The non-linear associations of biological age acceleration with hospital-diagnosed MASLD according to restricted cubic spline based on multiple-variable adjusted Cox proportional hazard regression models. CI, confidence interval; HR, hazard ratio; MASLD, Metabolic dysfunction-associated steatotic liver disease. The restricted cubic spline models used four knots at the 5th, 35th, 65th, and 95th. HRs and corresponding 95% CIs were estimated using the median value as the reference (PhenoAge acceleration: -0.6829; KDMAge acceleration: -1.5871). Solid lines represent HRs, and shaded areas indicate 95% CIs. Covariates adjusted include age (continuous, year), sex (male and female), race (White ethnicity or race and Others), education (less than high school, high school or equivalent, and college and above), Townsend deprivation index, body mass index (continuous, kg/m^2^), smoking status (never, previous, and current), alcohol consumption (never, previous, and current), physical activity (low, moderate, and high), and healthy diet (yes and no).

In unadjusted cumulative incidence curves, higher PhenoAge acceleration and KDMAge acceleration corresponded to increased incidence of hospital-diagnosed MASLD (both *P* values for log-rank test < 0.001; **Supplementary Figure 4**). When biological age accelerations were modeled as continuous variables, per SD increase in biological age acceleration was associated with incident hospital-diagnosed MASLD, with estimated HRs of 1.03 (95% CI: 1.03, 1.04) for PhenoAge acceleration and 1.03 (1.02, 1.05) for KDMAge acceleration, respectively. Meanwhile, people in the highest tertile group of PhenoAge acceleration showed a 62% higher risk of hospital-diagnosed MASLD, as compared to those in the lowest tertile group (1.62; 1.46, 1.79). For KDMAge acceleration, the HR was 1.51 (1.32, 1.74) for extreme comparison. Compared to participants with non-accelerated aging, those with accelerated aging, defined by PhenoAge acceleration and KDMAge acceleration, showed 46% (1.46; 1.35, 1.57) and 35% (1.35; 1.19, 1.53) increased risk of incident hospital-diagnosed MASLD, respectively. Further adjustment for hypertension, diabetes, and dyslipidemia in sensitivity analyses modestly attenuated the associations, although the results remained statistically significant (**Table 2**). These findings suggest that accelerated biological aging may identify individuals at elevated metabolic risk for clinically recognized MASLD. The restricted cubic splines suggested that biological age acceleration showed non-linear associations with incident hospital-diagnosed MASLD (overall *P* < 0.001 for both; *P* for non-linearity < 0.001 for PhenoAge acceleration and = 0.015 for KDMAge acceleration; **Figure 1**).

**Table 2:** Associations of the biological age accelerations with incident hospital-diagnosed MASLD at follow-up (N = 247,444).

Biological aging	Cases/participants	Model 1		Model 2		Model 2 + metabolic comorbidities^*^	
		HR (95% CI)	*P* value	HR (95% CI)	*P* value	HR (95% CI)	*P* value
PhenoAge acceleration (continuous)	3,254/247,444	1.06 (1.06, 1.06)	<0.001	1.03 (1.03, 1.04)	<0.001	1.03 (1.02, 1.03)	<0.001
PhenoAge acceleration (Tertiles)							
T1	584/81,657	1.00 (ref)		1.00 (ref)		1.00 (ref)	
T2	909/81,656	1.51 (1.36, 1.67)	<0.001	1.22 (1.10, 1.35)	<0.001	1.22 (1.10, 1.35)	<0.001
T3	1,761/84,131	2.67 (2.43, 2.93)	<0.001	1.62 (1.46, 1.79)	<0.001	1.54 (1.40, 1.71)	<0.001
Non-accelerated aging	1,152/137,927	1.00 (ref)		1.00 (ref)		1.00 (ref)	
Accelerated aging	2,102/109,517	2.13 (1.98, 2.29)	<0.001	1.46 (1.35, 1.57)	<0.001	1.40 (1.30, 1.52)	<0.001
KDMAge acceleration (continuous)	3,254/247,444	1.08 (1.07, 1.09)	<0.001	1.03 (1.02, 1.05)	<0.001	1.03 (1.02, 1.04)	<0.001
KDMAge acceleration (Tertiles)							
T1	696/81,657	1.00 (ref)		1.00 (ref)		1.00 (ref)	
T2	1,152/81,656	1.55 (1.40, 1.72)	<0.001	1.22 (1.10, 1.35)	<0.001	1.20 (1.07, 1.33)	0.001
T3	1,406/84,131	2.41 (2.10, 2.77)	<0.001	1.51 (1.32, 1.74)	<0.001	1.43 (1.24, 1.65)	<0.001
Non-accelerated aging	1,866/164,687	1.00 (ref)		1.00 (ref)		1.00 (ref)	
Accelerated aging	1,388/82,757	1.91 (1.69, 2.17)	<0.001	1.35 (1.19, 1.53)	<0.001	1.28 (1.13, 1.45)	<0.001

CI, confidence interval; HR, hazard ratio; KDM, Klemera-Doubal method; MASLD, metabolic dysfunction-associated steatotic liver disease.

Model 1: adjusted for age (continuous, year), sex (male and female), race and ethnicity (White ethnicity or race and Others), education (less than high school, high school or equivalent, and college and above), and Townsend deprivation index (continuous);

Model 2: Adjusted for body mass index (continuous, kg/m^2^), smoking status (never, previous, and current), alcohol consumption (never, previous, and current), physical activity (low, moderate, and high), healthy diet (yes and no), and covariates adjusted in Model 1.

^*^ Metabolic comorbidities include hypertension (yes and no), diabetes (yes and no), and dyslipidemia (yes and no).

**Figure 2:**
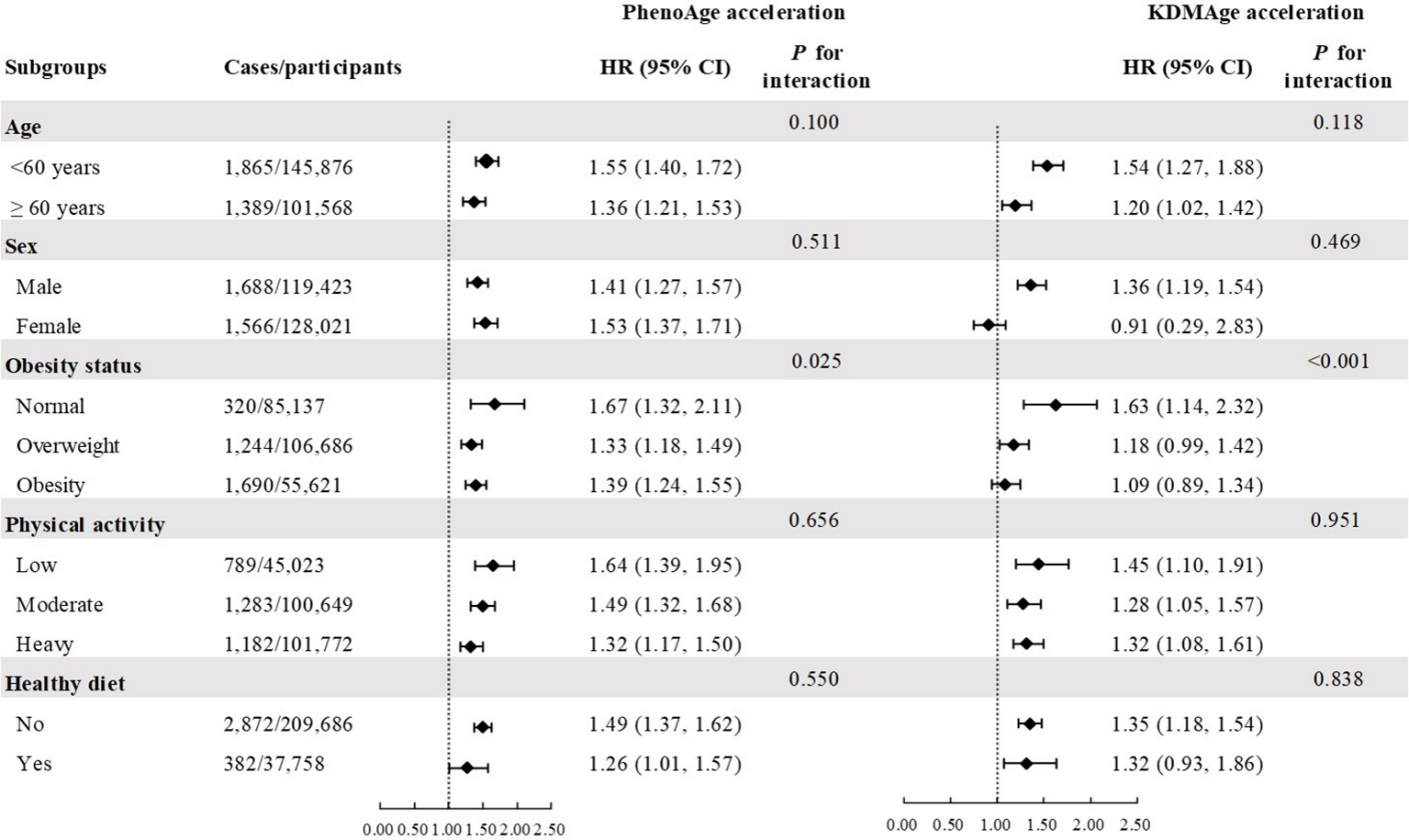
Subgroup analysis of the association of age acceleration with hospital-diagnosed MASLD. CI, confidence interval; HR, hazard ratio; KDM, Klemera-Doubal method; MASLD, Metabolic dysfunction-associated steatotic liver disease. Models were adjusted for age (continuous, year), sex (male and female), race (White ethnicity or race and Others), education (less than high school, high school or equivalent, and college and above), Townsend deprivation index (continuous), body mass index (continuous, kg/m^2^), smoking status (never, previous, and current), alcohol consumption (never, previous, and current), physical activity (low, moderate, and high), and healthy diet (yes and no). The HRs were estimated for accelerated aging in comparison with non-accelerated aging defined by PhenoAge residual and KDMAge residual. *P* for interaction was estimated using the Likelihood ratio test by comparing fully adjusted models with and without a multiplicative interaction term of age acceleration with a stratified factor. The analyses were conducted using Cox proportional hazard regression models.

In the exploratory subgroup analyses, the association between biological aging and hospital-diagnosed MASLD was stronger in people with normal weight (*P* value for interaction = 0.025 for PhenoAge acceleration and <0.001 for KDMAge acceleration; **Figure 2**). No significant interactions were observed between biological aging and other stratified factors. However, these subgroup findings should be interpreted cautiously, and the underlying reasons for these differences remain unclear.

Several sensitivity analyses were conducted to examine the robustness of the primary findings. In the cross-sectional analyses of biological aging and MASLD defined by PDFF ≥5% or FLI ≥60 together with cardiometabolic risk factors and low alcohol consumption, biological aging remained positively associated with MASLD (**Supplementary Table 6**). In addition, excluding participants who developed hospital-diagnosed MASLD within the first 5 years of follow-up or those with excessive alcohol consumption yielded effect estimates that were comparable in direction and magnitude to the primary analyses (**Supplementary Tables 7**, **8**). Similar associations were also observed after multiple imputation for missing covariates (**Supplementary Tables 9**, **10**).

### Associations between biological aging and cardiometabolic biomarkers

For this analysis, we further excluded participants with missing data on cardiometabolic biomarkers. Therefore, 242,834 participants were included. The distribution of cardiometabolic biomarkers is presented in **Supplementary Table 11**. PhenoAge acceleration showed negative associations with LDL-C, HDL-C, and TC (all *P* values < 0.001) and positive associations with other biomarkers except for TG (*P* = 0.561). For KDMAge acceleration, we noted negative associations between KDMAge acceleration and HDL-C (*P* value < 0.001). Meanwhile, KDMAge was positively associated with other biomarkers (**Supplementary Table 12**).

### Predictive ability of biological age acceleration and chronological age for hospital-diagnosed MASLD risk

We predicted the 8-year hospital-diagnosed MASLD risk and found that PhenoAge acceleration and KDMAge acceleration demonstrated better predictive performance than chronological age (AUROC: 0.653 vs. 0.524 and 0.611 vs. 0.524, both *P* values < 0.001). The AUROC of the base model comprised of BMI, AST, and ALT was 0.776. When we further included the PhenoAge acceleration and KDMAge acceleration into the base model, only PhenoAge acceleration yielded a significant increase in the AUROC (AUROC: 0.784 vs 0.776; *P* = 0.003), despite the small difference (**Supplementary Figure 5**).

### Potential mediation role of biological age acceleration in the association between lifestyles and hospital-diagnosed MASLD

Cox regression results showed that current smoking, drinking, poor diet, and low physical activity were positively associated with the risk of hospital-diagnosed MASLD, with adjusted HRs of 1.44 (1.29, 1.58), 1.18 (1.01, 1.40), 1.17 (1.05, 1.31), and 1.09 (1.00, 1.18), respectively (**Supplementary Table 13**). Furthermore, we also observed significant associations between these lifestyle factors and biological age acceleration (**Supplementary Table 14**). Exploratory mediation analyses demonstrated that PhenoAge acceleration significantly accounted for the associations between unhealthy lifestyle factors and MASLD. The mediation proportions were 25.5% (18.4%, 32.6%) for current smoking, 11.4% (1.1%, 21.8%) for drinking, 11.6% (4.0%, 19.1%) for poor diet, and 17.7% (1.1%, 34.3%) for low physical activity. By comparison, KDMAge acceleration showed relatively weak mediation effects, with mediation proportions of 1.9% (0.9%, 2.9%) for current smoking and 1.8% (0.3%, 2.5%) for poor diet (**Table 3**). As all variables were assessed at baseline, these findings should be interpreted as exploratory and do not imply causal mediation relationships.

**Table 3:** Mediation roles of biological age in the associations between life behaviors and incident hospital-diagnosed MASLD (N = 247,444).

Life behaviors	Direct association HR (95% CI)	Indirect association HR (95% CI)	Total association HR (95% CI)	Mediation proportion % (95% CI)
PhenoAge acceleration				
Current smoking	1.351 (1.221, 1.495)	1.089 (1.071, 1.107)	1.471 (1.330, 1.627)	25.5 (18.4, 32.6)
Drinking	1.169 (0.992, 1.378)	1.019 (1.013, 1.025)	1.191 (1.010, 1.403)	11.4 (1.1, 21.8)
Poor diet	1.156 (1.038, 1.288)	1.018 (1.013, 1.022)	1.177 (1.057, 1.310)	11.6 (4.0, 19.1)
Low physical activity	1.074 (0.990, 1.166)	1.015 (1.011, 1.019)	1.090 (1.004, 1.184)	17.7 (1.1, 34.3)
KDMAge acceleration				
Current smoking	1.425 (1.288, 1.575)	1.006 (1.003, 1.009)	1.433 (1.296, 1.584)	1.9 (0.9, 2.9)
Drinking	1.182 (1.003, 1.393)	1.001 (1.000, 1.002)	1.183 (1.004, 1.394)	0.5 (-0.3, 1.2)
Poor diet	1.166 (1.047, 1.299)	1.003 (1.001, 1.004)	1.169 (1.050, 1.302)	1.8 (0.3, 2.5)
Low physical activity	1.088 (1.002, 1.182)	1.000 (1.000, 1.001)	1.089 (1.003, 1.182)	0.6 (-0.2, 1.3)

BMI, body mass index; CI, confidence interval; HR, hazard ratio; MASLD, metabolic dysfunction-associated steatotic liver disease.

Models were adjusted for age (continuous, year), sex (male and female), race and ethnicity (White ethnicity or race and Others), education (less than high school, high school or equivalent, and college and above), Townsend deprivation index (continuous), body mass index (continuous, kg/m^2^), current smoking status (yes and no), alcohol consumption (never, previous, and current), physical activity (low, moderate, and heavy), and healthy diet (yes and no).

## Discussion

As one of the largest prospective studies on this topic, we found that accelerated biological aging was associated with a higher risk of hospital-diagnosed MASLD during follow-up. Furthermore, accelerated biological aging partly accounted for the associations of unhealthy lifestyles with hospital-diagnosed MASLD. These findings suggest that accelerated biological aging may be a useful marker for identifying populations at higher risk of hospital-diagnosed MASLD and may provide insights into the biological processes related to MASLD.

In this prospective study consisting of 247,444 participants with a median follow-up of 13.7 years, we provide convincing evidence that the risk of hospital-diagnosed MASLD is positively associated with biological aging, assessed by PhenoAge and KDMAge. Our findings are largely aligned with previous studies using various phenotypic ages, such as telomere length, epigenetic clocks, and other “aging clocks” derived from transcriptomic, proteomic, and metabolomic data. A prospective study of 467,848 adults from the UK Biobank evaluated the association between telomere length, a widely used biomarker of senescence, and the risk of MASLD, which revealed that long telomere length was associated with a decreased risk of MASLD (18). Another study based on the US NHANES demonstrated an inverse association between telomere length and prevalent MASLD only among Mexican-American men (31). DNA methylation age is another widely acknowledged proxy of biological age that could be modified by environmental and lifestyle factors. Epidemiological findings from the Shanghai Changfeng Study demonstrated that the risk of MASLD was nearly five times higher among individuals in the highest tertile group compared to those in the lowest tertile group of DNA methylation age acceleration (19). Furthermore, several studies have analyzed the association between biological aging and MASLD progression, and shorter telomere length was reported to be closely related to fibrosis (32, 33). These findings collectively support a potential link between biological aging and MASLD. However, the methods used to measure biological aging in these studies were usually complex, which poses a challenge in the standardization of detection. In contrast, the proxy for biological age used in our study is relatively simple and feasible for large population-based epidemiological studies. Moreover, PhenoAge and KDMAge incorporate information from multiple physiological systems and may therefore better identify individuals with accelerated biological aging. Our findings further support the association between biological aging and hospital-diagnosed MASLD and suggest that these measures may help identify populations at higher risk of hospital-diagnosed MASLD.

In the exploratory analyses, we observed stronger associations of biological age acceleration with hospital-diagnosed MASLD among participants with normal weight at the baseline survey. This finding is unexpected since overweight/obesity is identified risk factors for MASLD (34). While the precise mechanisms driving the heterogeneities among these subgroups remain difficult to decipher, insights from previous studies on environmental exposure may offer some clues. In one study examining the association between environmental polycyclic aromatic hydrocarbon exposure and diabetes among the Chinese population, the authors also found more pronounced associations in the subgroup of normal weight (35). Similarly, there is also evidence that people with a low-risk Framingham score had a greater reduction in heart rate variability response to PAH exposure (36). However, the reasons underlying these subgroup differences remain unclear. It is possible that the influence of biological aging may be more readily observed among individuals with fewer established cardiometabolic risk factors, although the current study was not designed to investigate lean MASLD-specific mechanisms. Therefore, these exploratory findings should be interpreted cautiously.

Several biological mechanisms may help explain the observed associations between biological aging and hospital-diagnosed MASLD. The biological aging measures used in this study incorporate biomarkers related to systemic inflammation, metabolic dysfunction, and organ function. Specifically, PhenoAge includes markers such as CRP, glucose, RDW, and white blood cell count, which reflect chronic inflammation and metabolic dysregulation closely involved in MASLD development. KDMAge additionally incorporates indicators related to cardiometabolic, renal, and pulmonary function, including systolic blood pressure, HbA1c, creatinine, blood urea nitrogen, and FEV1, thereby reflecting broader multisystem physiological status. The observed associations may therefore partly reflect the cumulative burden of inflammation-, metabolism-, and organ function-related physiological dysregulation involved in MASLD pathogenesis. Aging-related physiological changes may further contribute to MASLD development through impaired glucose and lipid metabolism (37) and reduced mitochondrial fatty acid β-oxidation (38, 39), which can promote hepatic lipid accumulation.

In our study, we observed that biological aging was positively associated with a series of cardiometabolic biomarkers, including biomarkers of glucose homeostasis, blood pressure, inflammation, and liver function. For lipid profiles, biological aging was negatively associated with HDL-C, while no consistent associations were observed for LDL-C and TC. PhenoAge acceleration was inversely associated with LDL-C and total cholesterol, whereas KDMAge acceleration showed positive associations with these lipid measures. These divergent patterns may be related to the cross-sectional nature of the study and the possibility of residual confounding. Moreover, after adding biological aging measures to prediction models including traditional risk factors, we observed a statistically significant but modest improvement in AUROC. Established MASLD-related prediction models, such as the FLI, Hepatic Steatosis Index (HSI), and NAFLD Fibrosis Score (NFS), mainly rely on liver enzymes and metabolic parameters, whereas biological aging measures may capture broader inflammation- and metabolism-related physiological dysregulation. Therefore, biological aging may provide complementary information regarding MASLD susceptibility. Nevertheless, the modest improvement in predictive performance observed in our study should be interpreted cautiously, and further studies are needed to evaluate its incremental clinical utility beyond established MASLD prediction models.

Smoking, drinking, poor diet, and inactivity are recognized as modifiable risk factors for MASLD development, but the underlying mechanisms remain incompletely understood. Using data from the UK Biobank, we found that PhenoAge may partly account for the associations between these lifestyle factors and hospital-diagnosed MASLD, with mediation proportions ranging from 11.4% to 25.5%. The indirect effects showed relatively narrower CIs than the corresponding direct effects. This difference should be interpreted cautiously because direct and indirect effects represent different components of the total association and are not directly comparable based solely on CI width on the HR scale. The relatively narrow CIs of the indirect effects may partly reflect their smaller effect sizes, together with the stable estimation of the exposure-mediator and mediator-outcome pathways in the large cohort. In contrast, direct effects represent the residual associations after accounting for the mediator, reflecting the remaining association after partitioning the total association and potentially resulting in wider CIs relative to the magnitude of the estimated effect. These findings suggest that accelerated biological aging may represent one pathway linking unhealthy lifestyles to hospital-diagnosed MASLD. This observation may help identify individuals with increased physiological vulnerability to lifestyle-related metabolic disease. Notably, the mediation effects were stronger for PhenoAge acceleration than for KDMAge acceleration, suggesting that different biological aging measures may capture partially distinct aging domains. Compared with KDMAge, PhenoAge incorporates biomarkers more closely related to systemic inflammation and metabolic dysfunction, which are central to MASLD pathogenesis. These findings suggest that inflammation- and metabolism-related biological aging may play a more important role in linking unhealthy lifestyles to MASLD risk. However, given the observational design and the simultaneous assessment of lifestyle factors and biological aging at baseline, the mediation findings should be interpreted cautiously and do not imply causal pathways. Further studies are needed to clarify the temporal relationships and underlying biological mechanisms.

The most notable strengths of our study include the large sample size and prospective design. However, there are still several limitations. First, biological aging was assessed at a single time point; thus, we could not evaluate how longitudinal changes in biological aging affect the hospital-diagnosed MASLD risk. Second, hospital-diagnosed MASLD was ascertained based on hospital records in the UK Biobank study, which may not fully align with the current MASLD diagnostic criteria and could lead to misclassification, particularly for early or undiagnosed cases. In addition, the relatively low incidence observed in our study likely reflects that this approach primarily captures clinically recognized cases, typically those severe enough to result in hospital contact, rather than all incident MASLD cases in the general population. Participants with poorer biological health may also have more frequent healthcare encounters and therefore a higher likelihood of receiving a hospital diagnosis of MASLD, which could introduce detection bias. Although we excluded participants with known liver diseases at baseline, undiagnosed MASLD may still have been present and could potentially contribute to reverse causation. However, this approach has been widely used in UK Biobank studies, and we excluded participants with other known causes of liver disease to improve specificity. Moreover, in sensitivity analyses using the recently proposed MASLD criteria, the overall association patterns remained consistent despite the cross-sectional design. This finding suggests that the associations between biological aging and fatty liver disease were broadly consistent under the updated MASLD framework. Third, because of missing values in the covariates, only a set of subjects were included in the primary analyses, which might induce selection bias. To address this limitation, we performed multiple imputations for missing covariates during the sensitivity analysis and observed no significant changes in the results. Fourth, mediation analyses were based on variables measured at baseline, and therefore the temporal relationships among lifestyle factors, biological aging, and hospital-diagnosed MASLD could not be fully established. Fifth, as an observational study, we cannot infer causality, and residual confounding cannot be entirely excluded. Sixth, race/ethnicity was categorized as White versus others, which may not fully capture potential heterogeneity across different ethnic groups. Therefore, caution is warranted when generalizing our findings to more diverse populations. Finally, the biological aging measures used in this study (PhenoAge and KDMAge) incorporate biomarkers related to metabolic function, inflammation, and organ function. Therefore, some overlap with underlying metabolic abnormalities and disease processes cannot be excluded. However, these measures are composite indices intended to capture multisystem physiological dysregulation beyond isolated metabolic alterations. Accordingly, the observed associations likely reflect integrated biological aging processes rather than metabolic abnormalities alone. Moreover, although these measures may help identify populations at higher risk of hospital-diagnosed MASLD, external validation of their predictive performance for MASLD is still needed.

In conclusion, in this prospective study, accelerated biological aging is associated with a higher risk of hospital-diagnosed MASLD. In addition, biological aging may partly explain the associations of unhealthy lifestyles with hospital-diagnosed MASLD. These findings provide further evidence supporting the potential relevance of biological aging in MASLD development and highlight the importance of considering age-related processes in future MASLD research. Further studies in more diverse populations and with more comprehensive phenotyping are needed to validate these findings and better clarify the relationships between biological aging and MASLD.

## Data Availability

The data analyzed in this study are available through application to the UK Biobank and can be accessed by qualified researchers following approved research proposal. Requests to access the datasets should be directed to https://www.ukbiobank.ac.uk.
