# Supplementary material for: Prospective association between biological aging and risk of hospital-diagnosed MASLD: evidence from the UK Biobank
Source: Front Endocrinol (Lausanne). 2026 Jun 17;17:1853319. doi: 10.3389/fendo.2026.1853319 (PMC13318727; doi:10.3389/fendo.2026.1853319)
Supplement: Supplementary file 1 [file DataSheet1.docx]

**Supplementary Materials for**

**Prospective association between biological aging and risk of hospital-diagnosed MASLD: Evidence from the UK Biobank**

**Contents**

[Supplementary Methods 1](#_Toc20206)

[Supplementary Table 1 Variables used for the calculation of PhenoAge and KDMAge in the UK Biobank. 3](#_Toc707)

[Supplementary Table 2 Criteria for the liver disease at baseline in UK Biobank. 4](#_Toc23950)

[Supplementary Table 3 Baseline characteristics of participants from UK Biobank 7](#_Toc16061)

[Supplementary Table 4 Baseline characteristics of participants included and excluded in final analysis 9](#_Toc2350)

[Supplementary Table 5 Baseline characteristics of participants with and without PDFF measures 10](#_Toc22481)

[Supplementary Table 6 Associations of the biological age accelerations with MASLD defined by PDFF (N=22,370) and FLI (N=247,347) at baseline 11](#_Toc14895)

[Supplementary Table 7 Associations of biological aging with incident hospital-diagnosed MASLD: Exclude MASLD cases diagnosed within the first 5 years of follow-up (N = 246,560) 13](#_Toc24498)

[Supplementary Table 8 Associations of biological aging with incident hospital-diagnosed MASLD: Exclude excessive drinkers 14](#_Toc8968)

[Supplementary Table 9 Summary of missing data of covariates for multiple imputation 15](#_Toc12373)

[Supplementary Table 10 Associations of biological aging with incident hospital-diagnosed MASLD using the imputed dataset 16](#_Toc27056)

[Supplementary Table 11 Cardiometabolic biomarkers at baseline survey 17](#_Toc9295)

[Supplementary Table 12 Associations between biological aging and cardiometabolic biomarkers 18](#_Toc24849)

[Supplementary Table 13 Associations of lifestyles with incident hospital-diagnosed MASLD during follow-up 19](#_Toc17117)

[Supplementary Table 14 Associations of lifestyles with biological age acceleration 20](#_Toc31597)

[Supplementary Figure 1 Flowchart of selection of participants in the UK Biobank. 21](#_Toc1361)

[Supplementary Figure 2 Directed acyclic graph illustrating the hypothesized causal relationships between biological aging and incident MASLD. 22](#_Toc9375)

[Supplementary Figure 3 The distribution of biological age and age acceleration across non-MASLD and hospital-diagnosed MASLD. 23](#_Toc18615)

[Supplementary Figure 4 Kaplan-Meier survival estimates according to biological age and age acceleration for the probability of incident hospital-diagnosed MASLD in UK Biobank. 24](#_Toc27542)

[Supplementary Figure 5 Receiver-operating characteristic curves for 8-year hospital-diagnosed MASLD risk 25](#_Toc22009)

# Supplementary Methods

**Calculation of biological aging measures**

PhenoAge was calculated based on chronological age and nine clinical biomarkers using the algorithm developed by Levine et al. These biomarkers included albumin, creatinine, glucose, C-reactive protein (CRP), lymphocyte percentage (lymph), mean cell volume (MCV), red cell distribution width (RDW), alkaline phosphatase (ALP), and white blood cell count (WBC). The calculation was implemented using the BioAge R package, and the formula can be expressed as follows:

xb = -19.90667 - 0.03359355 × albumin + 0.009506491 × creatinine + 0.1953192 × glucose +

0.09536762 × ln(CRP) - 0.01199984 × lymph + 0.02676401 × MCV + 0.3306156 × RDW +

0.001868778 × ALP + 0.05542406 × WBC + 0.08035356 × age

m = 1 - exp(-1.51714 × exp(xb)) / 0.007692696

PhenoAge = ln(-0.0055305 × ln(1 - m)) / 0.090165 + 141.50225

KDMAge was calculated using the Klemera–Doubal method based on chronological age and a set of clinical biomarkers, including albumin, creatinine, CRP, alkaline phosphatase, blood urea nitrogen, HbA1c, total cholesterol, systolic blood pressure, and forced expiratory volume in one second (FEV1). The biological age estimate was calculated as:

$$KDMAge=\frac{\sum_{j=1}^{m} \left( x_{j}-q_{j} \right)\frac{k_{j}}{s_{j}^{2}}+\frac{age}{s_{age}^{2}}}{\sum_{j=1}^{m} \left( \frac{k_{j}}{s_{j}} \right)^{2}+\frac{1}{s_{age}^{2}}}$$

Where *x_j_* represents the observed value of biomarker *j*, and *q_j_*, *k_j_*, and *s_j_* denote the intercept, slope, and residual standard deviation from the regression of each biomarker on chronological age, respectively. The calculation was implemented using the BioAge R package, following the original published algorithm.

All variables were obtained from the UK Biobank database using the corresponding field IDs (Supplementary Table 1). Unit conversions were performed where necessary to ensure consistency with the original algorithms. Details of units and conversion procedures are provided in Supplementary Table 1.

**Definition of MASLD based on fatty liver index (FLI)**

MASLD was further defined according to the consensus criteria proposed by Rinella et al. (Rinella et al., 2023). Hepatic steatosis was identified using the fatty liver index (FLI), and an FLI ≥60 was considered indicative of steatosis. FLI was calculated using body mass index (BMI), waist circumference, triglyceride levels, and gamma-glutamyl transferase levels. In addition to hepatic steatosis, participants were required to have at least one cardiometabolic risk factor and low alcohol consumption (<20 g/day for women and <30 g/day for men). Cardiometabolic risk factors included: (1) overweight/obesity, defined as body mass index ≥25 kg/m² and/or waist circumference >94 cm in men or >90 cm in women; (2) diabetes, defined as glycated hemoglobin (HbA1c) ≥48 mmol/mol, or diagnosis of type 2 diabetes, or treatment for type 2 diabetes; (3) hypertension, defined as systolic blood pressure ≥130 mmHg, or diastolic blood pressure ≥85 mmHg, or diagnosis of hypertension, or use of antihypertensive medication; (4) hypertriglyceridemia, defined as triglycerides ≥1.70 mmol/L or use of lipid-lowering medication; and (5) low high-density lipoprotein (HDL) cholesterol, defined as HDL cholesterol ≤1.0 mmol/L in men or ≤1.3 mmol/L in women, or use of lipid-lowering medication (Feng, Qi et al., 2025) .

# Supplementary Table 1 Variables used for the calculation of PhenoAge and KDMAge in the UK Biobank.

| **Variable** | **Algorithm** | **UK Biobank field ID** | **Unit (UKB)** | **Unit (algorithm)** | **Conversion applied** |
| --- | --- | --- | --- | --- | --- |
| Chronological age | Both | 21022 | years | years | None |
| Albumin | Both | 30600 | g/L | g/L | None |
| Creatinine | Both | 30700 | µmol/L | µmol/L | None |
| C-reactive protein (CRP) | Both | 30710 | mg/L | mg/dL | ÷10 |
| Alkaline phosphatase (ALP) | Both | 30610 | U/L | U/L | None |
| White blood cell count (WBC) | PhenoAge | 30000 | 10⁹ cells/L | 1000 cells/µL | Equivalent (no conversion needed) |
| Lymphocyte (%) | PhenoAge | 30180 | % | % | None |
| Mean cell volume (MCV) | PhenoAge | 30270 | fL | fL | None |
| Red cell distribution width (RDW) | PhenoAge | 30070 | % | % | None |
| Glucose | PhenoAge | 30740 | mmol/L | mmol/L | None |
| Blood urea nitrogen (BUN) | KDMAge | 30670 | mmol/L | mg/dL | ×2.8 |
| HbA1c | KDMAge | 30750 | mmol/mol | % | ×0.0915 + 2.15 |
| Total cholesterol | KDMAge | 30690 | mmol/L | mg/dL | ×38.665 |
| Systolic blood pressure (SBP) | KDMAge | 4080 | mmHg | mmHg | None |
| Forced expiratory volume (FEV1) | KDMAge | 3063 | L | L | None |

Abbreviation: HbA1c, glycosylated hemoglobin; UKB, UK Biobank.

# Supplementary Table 2 Criteria for the liver disease at baseline in UK Biobank.

| **ICD-10 (data field 41270)** | | **ICD-9 (data field 41270)** | | **Self-reported (data field 20002)** | |
| --- | --- | --- | --- | --- | --- |
| **Code** | **Description** | **Code** | **Description** | **Code** | **Description** |
| K76.0 | Fatty (change of) liver, not elsewhere classified | 5710 | Alcoholic fatty liver | 1136 | Liver/biliary/pancreas problem |
| K75.8 | Other specified inflammatory liver diseases | 5711 | Acute alcoholic hepatitis | 1141 | Oesophageal varices |
| K70.0 | Alcoholic fatty liver | 5712 | Alcoholic cirrhosis of liver | 1155 | Hepatitis |
| K70.1 | Alcoholic hepatitis | 5713 | Alcoholic liver damage, unspecified | 1156 | Infective/viral hepatitis |
| K70.2 | Alcoholic fibrosis and sclerosis of liver | 0701 | Viral hepatitis a without mention of hepatic coma | 1157 | Non-infective hepatitis |
| K70.3 | Alcoholic cirrhosis of liver | 0703 | Viral hepatitis b without mention of hepatic coma | 1158 | Liver failure/cirrhosis |
| K70.4 | Alcoholic hepatic failure | 0705 | Other specified viral hepatitis without mention of hepatic coma | 1159 | Bile duct disease |
| K70.9 | Alcoholic liver disease, unspecified | 0709 | Unspecified viral hepatitis without mention of hepatic coma | 1408 | Alcohol dependency |
| K74.0 | Hepatic fibrosis | 5731 | Hepatitis in viral diseases classified elsewhere | 1506 | Primary biliary cirrhosis |
| B15.9 | Hepatitis A without hepatic coma | 5715 | Cirrhosis of liver without mention of alcohol | 1507 | Haemochromatosis |
| B16.0 | Acute hepatitis B with delta-agent (coinfection) with hepatic coma | 5716 | Biliary cirrhosis | 1578 | Hepatitis a |
| B16.9 | Acute hepatitis B without delta-agent and without hepatic coma |  |  | 1579 | Hepatitis b |
| B17.0 | Acute delta-(super) infection of hepatitis B carrier |  |  | 1580 | Hepatitis c |
| B17.1 | Acute hepatitis C |  |  | 1581 | Hepatitis d |
| B17.2 | Acute hepatitis E |  |  | 1582 | Hepatitis e |
| B17.8 | Other specified acute viral hepatitis |  |  | 1604 | Alcoholic liver disease/alcoholic cirrhosis |
| B17.9 | Acute viral hepatitis, unspecified |  |  |  |  |
| B18.0 | Chronic viral hepatitis B with delta-agent |  |  |  |  |
| B18.1 | Chronic viral hepatitis B without delta-agent |  |  |  |  |
| B18.2 | Chronic viral hepatitis C |  |  |  |  |
| B18.8 | Other chronic viral hepatitis |  |  |  |  |
| B18.9 | Chronic viral hepatitis, unspecified |  |  |  |  |
| B19.9 | Unspecified viral hepatitis without coma |  |  |  |  |
| K74.0 | Hepatic fibrosis |  |  |  |  |
| K74.1 | Hepatic sclerosis |  |  |  |  |
| K74.2 | Hepatic fibrosis with hepatic sclerosis |  |  |  |  |
| K74.3 | Primary biliary cirrhosis |  |  |  |  |
| K74.4 | Secondary biliary cirrhosis |  |  |  |  |
| K74.5 | Biliary cirrhosis, unspecified |  |  |  |  |
| K74.6 | Other and unspecified cirrhosis of liver |  |  |  |  |
| K76.6 | Portal hypertension |  |  |  |  |
| K76.7 | Hepatorenal syndrome |  |  |  |  |
| I85.0 | Oesophageal varices with bleeding |  |  |  |  |
| I85.9 | Oesophageal varices without bleeding |  |  |  |  |
| I86.4 | Gastric varices |  |  |  |  |
| I98.2 | Oesophageal varices in diseases classified elsewhere |  |  |  |  |
| I98.3 | Oesophageal varices with bleeding in diseases classified elsewhere |  |  |  |  |
| R18 | Ascites |  |  |  |  |
| Z94.4 | Liver transplant |  |  |  |  |
| C22.0 | Liver cell carcinoma |  |  |  |  |

Supplementary Table 3 Baseline characteristics of participants from UK Biobank **(N = 247,444) ^*^**

| **Characteristics** | **Total (N = 247,444)** | **Non-accelerated aging (N = 137,927)** § | **Accelerated aging (N = 109,517)** § | ***P*** values |
| --- | --- | --- | --- | --- |
| **Chronological age, years** | 56.08 (8.12) | 56.05 (8.12) | 56.11 (8.12) | 0.072 |
| **Sex** |  |  |  | <0.001 |
| Male | 119,423 (48.3%) | 59,770 (43.3%) | 59,653 (54.5%) |  |
| Female | 128,021 (51.7%) | 78,157 (56.7%) | 49,864 (45.5%) |  |
| **Race and ethnicity** |  |  |  | <0.001 |
| White ethnicity or race | 236,288 (95.5%) | 132,659 (96.2%) | 103,629 (94.6%) |  |
| Others | 11,156 (4.5%) | 5,268 (3.8%) | 5,888 (5.4%) |  |
| **Education** |  |  |  | <0.001 |
| Less than high school | 32,861 (13.3%) | 15,854 (11.5%) | 17,007 (15.5%) |  |
| High school or equivalent | 125,371 (50.7%) | 67,782 (49.1%) | 57,589 (52.6%) |  |
| College and above | 89,212 (36.1%) | 54,291 (39.4%) | 34,921 (31.9%) |  |
| **Townsend deprivation index** | -1.51 (2.96) | -1.70 (2.85) | -1.23 (3.09) |  |
| **Body mass index, kg/m**2 | 27.18 (4.58) | 26.02 (3.81) | 28.65 (5.04) |  |
| **Obesity status** |  |  |  | <0.001 |
| Normal | 85,137 (34.4%) | 59,388 (43.1%) | 25,749 (23.5%) |  |
| Overweight | 106,686 (43.1%) | 59,255 (43.0%) | 47,431 (43.3%) |  |
| Obesity | 55,621 (22.5%) | 19,284 (14.0%) | 36,337 (33.2%) |  |
| **Smoking status** |  |  |  | <0.001 |
| Never | 136,971 (55.4%) | 81,366 (59.0%) | 55,605 (50.8%) |  |
| Previous | 86,479 (34.9%) | 48,021 (34.8%) | 38,458 (35.1%) |  |
| Current | 23,994 (9.7%) | 8,540 (6.2%) | 15,454 (14.1%) |  |
| **Alcohol consumption** |  |  |  | <0.001 |
| Never | 9,045 (3.7%) | 4,549 (3.3%) | 4,496 (4.1%) |  |
| Previous | 7,736 (3.1%) | 3,743 (2.7%) | 3,993 (3.6%) |  |
| Current | 230,663 (93.2%) | 129,635 (94.0%) | 101,028 (92.2%) |  |
| **Physical activity** |  |  |  | <0.001 |
| Low | 45,023 (18.2%) | 21,941 (15.9%) | 23,082 (21.1%) |  |
| Moderate | 100,649 (40.7%) | 56,447 (40.9%) | 44,202 (40.4%) |  |
| High | 101,772 (41.1%) | 59,539 (43.2%) | 42,233 (38.6%) |  |
| **Healthy diet**, yes | 37,758 (15.3%) | 23,668 (17.2%) | 14,090 (12.9%) | <0.001 |
| **Hypertension, yes** | 121,271 (49.0%) | 61,870 (44.9%) | 59,401 (54.2%) | <0.001 |
| **Diabetes, yes** | 10,285 (4.2%) | 2,546 (1.8%) | 7,739 (7.1%) | <0.001 |
| **Dyslipidemia, yes** | 132,767 (53.7%) | 73,423 (53.2%) | 59,344 (54.2%) | <0.001 |
| **Components of biological ages** |  |  |  |  |
| FEV1, L | 2.79 (0.79) | 2.82 (0.79) | 2.75 (0.79) | <0.001 |
| SBP, mm Hg | 137.20 (18.38) | 136.01 (18.40) | 138.77 (18.24) | <0.001 |
| Total cholesterol, mg/dL | 220.48 (43.52) | 222.81 (42.73) | 217.55 (44.33) | <0.001 |
| HbA1c, % | 5.42 (0.58) | 5.32 (0.40) | 5.56 (0.72) | <0.001 |
| Blood urea nitrogen, mg/dL | 15.09 (3.76) | 14.79 (3.33) | 15.46 (4.21) | <0.001 |
| Lymphocyte, % | 29.00 (7.38) | 30.71 (7.01) | 26.84 (7.26) | <0.001 |
| Mean cell volume, fL | 82.79 (5.28) | 82.01 (4.79) | 83.76 (5.69) | <0.001 |
| Serum glucose, mmol/L | 5.09 (1.16) | 4.91 (0.64) | 5.32 (1.56) | <0.001 |
| Red cell distribution width, % | 13.45 (0.94) | 13.12 (0.58) | 13.87 (1.12) | <0.001 |
| White cell count, 1000 cells/µL | 6.82 (1.89) | 6.21 (1.36) | 7.58 (2.18) | <0.001 |
| Albumin, g/L | 4.53 (0.26) | 4.58 (0.25) | 4.47 (0.26) | <0.001 |
| Creatinine, mg/dL | 0.82 (0.18) | 0.79 (0.14) | 0.87 (0.21) | <0.001 |
| C-reactive protein, mg/dL | 0.24 (0.39) | 0.11 (0.12) | 0.39 (0.54) | <0.001 |
| Alkaline phosphatase, U/L | 82.18 (25.08) | 77.60 (20.38) | 87.96 (28.94) | <0.001 |

|  |
| --- |

Abbreviation: FEV1, forced expiratory volume in 1-second; HbA1c, glycosylated hemoglobin; SBP, systolic blood pressure.

^*^ Continuous variables were presented as mean (standard deviation) and categorical variables were presented as number (n, %). *P* values were calculated using the Student’s t-test and chi-square test for continuous and categorical characteristics, respectively.

^§^ Biological aging was defined by PhenoAge acceleration.

Supplementary Table 4 Baseline characteristics of participants included and excluded in final analysis **^*^**

| **Characteristics** | **Total  (N = 502,164)** | **Excluded  (N = 254,720)** | **Included  (N = 247,444)** | ***P* value** |
| --- | --- | --- | --- | --- |
| **Age, years** | 56.53 (8.09) | 56.97 (8.05) | 56.08 (8.12) | <0.001 |
| **Sex** |  |  |  | <0.001 |
| Male | 228,988 (45.6%) | 109,565 (43.0%) | 119,423 (48.3%) |  |
| Female | 273,176 (54.4%) | 145,155 (57.0%) | 128,021 (51.7%) |  |
| **Race and ethnicity** |  |  |  | <0.001 |
| White ethnicity or race | 472,392 (94.1%) | 236,104 (92.7%) | 236,288 (95.5%) |  |
| Others | 26,997 (5.4%) | 15,841 (6.2%) | 11,156 (4.5%) |  |
| **Education** |  |  |  | <0.001 |
| Less than high school | 85,235 (17.0%) | 52,374 (20.6%) | 32,861 (13.3%) |  |
| High school or equivalent | 245,791 (48.9%) | 120,420 (47.3%) | 125,371 (50.7%) |  |
| College and above | 161,016 (32.1%) | 71,804 (28.2%) | 89,212 (36.1%) |  |
| **Townsend deprivation index** | -1.29 (3.09) | -1.10 (3.20) | -1.50 (2.97) | <0.001 |
| **Body mass index, kg/m**2 | 27.43 (4.80) | 27.68 (5.00) | 27.18 (4.58) | <0.001 |
| **Obesity status** |  |  |  | <0.001 |
| Normal | 164,901 (32.8%) | 79,764 (31.3%) | 85,137 (34.4%) |  |
| Overweight | 211,980 (42.2%) | 105,294 (41.3%) | 106,686 (43.1%) |  |
| Obesity | 122,180 (24.3%) | 66,559 (26.1%) | 55,621 (22.5%) |  |
| **Smoking status** |  |  |  | <0.001 |
| Never | 273,344 (54.4%) | 136,373 (53.5%) | 136,971 (55.4%) |  |
| Previous | 172,935 (34.4%) | 86,456 (33.9%) | 86,479 (34.9%) |  |
| Current | 52,937 (10.5%) | 28,943 (11.4%) | 23,994 (9.7%) |  |
| **Alcohol consumption** |  |  |  | <0.001 |
| Never | 22,365 (4.5%) | 13,320 (5.2%) | 9,045 (3.7%) |  |
| Previous | 18,087 (3.6%) | 10,351 (4.1%) | 7,736 (3.1%) |  |
| Current | 460,060 (91.6%) | 229,397 (90.1%) | 230,663 (93.2%) |  |
| **Physical activity** |  |  |  | <0.001 |
| Low | 71,541 (14.2%) | 26,518 (10.4%) | 45,023 (18.2%) |  |
| Moderate | 156,453 (31.2%) | 55,804 (21.9%) | 100,649 (40.7%) |  |
| High | 157,034 (31.3%) | 55,262 (21.7%) | 101,772 (41.1%) |  |
| **Healthy diet, yes** | 72,736 (14.5%) | 34,978 (13.7%) | 37,758 (15.3%) | <0.001 |
| **Hypertension, yes** | 240,031 (47.8%) | 118,760 (46.6%) | 121,271 (49.0%) | <0.001 |
| **Diabetes, yes** | 24,036 (4.8%) | 13,751 (5.4%) | 10,285 (4.2%) | <0.001 |
| **Dyslipidemia, yes** | 234,602 (46.7%) | 101,835 (40.0%) | 132,767 (53.7%) | <0.001 |

^*^ Continuous variables were presented as mean ± standard deviation and categorical variables were presented as numbers (percentages). *P* values were calculated using the Student's t-test and chi-square test for continuous and categorical characteristics, respectively.

Supplementary Table 5 Baseline characteristics of participants with and without PDFF measures **^*^**

| **Characteristics** | **Total  (N = 247,444)** | **Without PDFF**  **(N = 225,074)** | **With PDFF**  **(N = 22,370)** |
| --- | --- | --- | --- |
| **Age, years** | 56.08 (8.12) | 56.20 (8.17) | 54.85 (7.53) |
| **Sex** |  |  |  |
| Male | 119,423 (48.3%) | 108,259 (48.1%) | 11,164 (49.9%) |
| Female | 128,021 (51.7%) | 116,815 (51.9%) | 11,206 (50.1%) |
| **Race and ethnicity** |  |  |  |
| White ethnicity or race | 236,288 (95.5%) | 214,548 (95.3%) | 21,740 (97.2%) |
| Others | 11,156 (4.5%) | 10,526 (4.7%) | 630 (2.8%) |
| **Education** |  |  |  |
| Less than high school | 32,861 (13.3%) | 31,664 (14.1%) | 1,197 (5.4%) |
| High school or equivalent | 125,371 (50.7%) | 115,022 (51.1%) | 10,349 (46.3%) |
| College and above | 89,212 (36.1%) | 78,388 (34.8%) | 10,824 (48.4%) |
| **Townsend deprivation index** | -1.51 (2.96) | -1.45 (2.99) | -1.92 (2.69) |
| **Body mass index, kg/m**2 | 27.18 (4.58) | 27.25 (4.62) | 26.49 (4.11) |
| **Obesity status** |  |  |  |
| Normal | 85,137 (34.4%) | 76,173 (33.8%) | 8,964 (40.1%) |
| Overweight | 106,686 (43.1%) | 97,140 (43.2%) | 9,546 (42.7%) |
| Obesity | 55,621 (22.5%) | 51,761 (23.0%) | 3,860 (17.3%) |
| **Smoking status** |  |  |  |
| Never | 136,971 (55.4%) | 123,341 (54.8%) | 13,630 (60.9%) |
| Previous | 86,479 (34.9%) | 79,109 (35.1%) | 7,370 (32.9%) |
| Current | 23,994 (9.7%) | 22,624 (10.1%) | 1,370 (6.1%) |
| **Alcohol consumption** |  |  |  |
| Never | 9,045 (3.7%) | 8,505 (3.8%) | 540 (2.4%) |
| Previous | 7,736 (3.1%) | 7,287 (3.2%) | 449 (2.0%) |
| Current | 230,663 (93.2%) | 209,282 (93.0%) | 21,381 (95.6%) |
| **Physical activity** |  |  |  |
| Low | 45,023 (18.2%) | 40,914 (18.2%) | 4,109 (18.4%) |
| Moderate | 100,649 (40.7%) | 91,241 (40.5%) | 9,408 (42.1%) |
| High | 101,772 (41.1%) | 92,919 (41.3%) | 8,853 (39.6%) |
| **Healthy diet, yes** | 37,758 (15.3%) | 34,169 (15.2%) | 3,589 (16.0%) |
| **Hypertension, yes** | 121,271 (49.0%) | 111,778 (49.7%) | 9,493 (42.4%) |
| **Diabetes, yes** | 10,285 (4.2%) | 9,760 (4.3%) | 525 (2.3%) |
| **Dyslipidemia, yes** | 132,767 (53.7%) | 120,963 (53.7%) | 11,804 (52.8%) |
| **PhenoAge acceleration, yes** | 109,517 (44.3%) | 100,945 (44.8%) | 8,572 (38.3%) |
| **KDMAge acceleration, yes** | 82,757 (33.4%) | 75,524 (33.6%) | 7,233 (32.3%) |

Abbreviation: PDFF, proton density fat fraction.

^*^Continuous variables were presented as mean (standard deviation) and categorical variables were presented as n (%). *P* values were calculated using the Student’s t-test and chi-square test for continuous and categorical characteristics, respectively.

Supplementary Table 6 Associations of the biological age accelerations with MASLD defined by PDFF (N=22,370) and FLI (N=247,347) at baseline

| **Biological aging** | **Model 1** | |  | **Model 2** | |  | **Model 2 + metabolic comorbidities^$^** | |
| --- | --- | --- | --- | --- | --- | --- | --- | --- |
|  | **OR (95% CI)** | ***P* value** |  | **OR (95% CI)** | ***P* value** |  | **OR (95% CI)** | ***P* value** |
| **MASLD defined by PDFF^*^** |  |  |  |  |  |  |  |  |
| PhenoAge acceleration (continuous) | 1.06 (1.06, 1.07) | <0.001 |  | 1.01 (1.00, 1.02) | 0.004 |  | 1.01 (1.00, 1.02) | 0.007 |
| PhenoAge acceleration (Tertiles) |  |  |  |  |  |  |  |  |
| T1 | 1.00 (ref) |  |  | 1.00 (ref) |  |  | 1.00 (ref) |  |
| T2 | 1.55 (1.44, 1.67) | <0.001 |  | 1.15 (1.06, 1.25) | <0.001 |  | 1.15 (1.06, 1.25) | <0.001 |
| T3 | 2.14 (1.99, 2.31) | <0.001 |  | 1.17 (1.08, 1.27) | <0.001 |  | 1.17 (1.07, 1.27) | <0.001 |
| Non-accelerated aging | 1.00 (ref) |  |  | 1.00 (ref) |  |  | 1.00 (ref) |  |
| Accelerated aging | 1.71 (1.61, 1.82) | <0.001 |  | 1.10 (1.03, 1.18) | 0.004 |  | 1.10 (1.03, 1.18) | 0.005 |
| KDMAge acceleration (continuous) | 1.12 (1.11, 1.14) | <0.001 |  | 1.06 (1.05, 1.07) | <0.001 |  | 1.04 (1.03, 1.05) | <0.001 |
| KDMAge acceleration (Tertiles) |  |  |  |  |  |  |  |  |
| T1 | 1.00 (ref) |  |  | 1.00 (ref) |  |  | 1.00 (ref) |  |
| T2 | 1.97 (1.81, 2.15) | <0.001 |  | 1.46 (1.33, 1.60) | <0.001 |  | 1.29 (1.17, 1.42) | <0.001 |
| T3 | 3.25 (2.94, 3.59) | <0.001 |  | 1.82 (1.64, 2.03) | <0.001 |  | 1.54 (1.38, 1.72) | <0.001 |
| Non-accelerated aging | 1.00 (ref) |  |  | 1.00 (ref) |  |  | 1.00 (ref) |  |
| Accelerated aging | 2.35 (2.15, 2.57) | <0.001 |  | 1.51 (1.38, 1.66) | <0.001 |  | 1.35 (1.22, 1.48) | <0.001 |
| **MASLD defined by FLI^#^** |  |  |  |  |  |  |  |  |
| PhenoAge acceleration (continuous) | 1.09 (1.09, 1.09) | <0.001 |  | 1.02 (1.02, 1.02) | <0.001 |  | 1.02 (1.01, 1.02) | <0.001 |
| PhenoAge acceleration (Tertiles) |  |  |  |  |  |  |  |  |
| T1 | 1.00 (ref) |  |  | 1.00 (ref) |  |  | 1.00 (ref) |  |
| T2 | 1.87 (1.82, 1.92) | <0.001 |  | 1.18 (1.15, 1.22) | <0.001 |  | 1.18 (1.14, 1.21) | <0.001 |
| T3 | 3.11 (3.04, 3.19) | <0.001 |  | 1.27 (1.23, 1.30) | <0.001 |  | 1.25 (1.21, 1.29) | <0.001 |
| Non-accelerated aging | 1.00 (ref) |  |  | 1.00 (ref) |  |  | 1.00 (ref) |  |
| Accelerated aging | 2.21 (2.17, 2.25) | <0.001 |  | 1.15 (1.13, 1.18) | <0.001 |  | 1.14 (1.12, 1.17) | <0.001 |
| KDMAge acceleration (continuous) | 1.13 (1.13, 1.13) | <0.001 |  | 1.04 (1.03, 1.04) | <0.001 |  | 1.03 (1.02, 1.03) | <0.001 |
| KDMAge acceleration (Tertiles) |  |  |  |  |  |  |  |  |
| T1 | 1.00 (ref) |  |  | 1.00 (ref) |  |  | 1.00 (ref) |  |
| T2 | 2.41 (2.23, 2.48) | <0.001 |  | 1.72 (1.67, 1.79) | <0.001 |  | 1.55 (1.50, 1.61) | <0.001 |
| T3 | 4.20 (4.06, 4.34) | <0.001 |  | 1.92 (1.85, 2.00) | <0.001 |  | 1.71 (1.65, 1.79) | <0.001 |
| Non-accelerated aging | 1.00 (ref) |  |  | 1.00 (ref) |  |  | 1.00 (ref) |  |
| Accelerated aging | 2.68 (2.60, 2.76) | <0.001 |  | 1.44 (1.40, 1.49) | <0.001 |  | 1.33 (1.29, 1.38) | <0.001 |

Abbreviation: CI, confidence interval; FLI. Fatty liver index; KDM, Klemera-Doubal method; MASLD, metabolic dysfunction-associated steatotic liver disease; OR, odds ratio; PDFF, proton density fat fraction.

Model 1: adjusted for age (continuous, year), sex (male and female), race and ethnicity (White ethnicity or race and Others), education (less than high school, high school or equivalent, and college and above), and Townsend deprivation index (continuous);

Model 2: Adjusted for body mass index (continuous, kg/m^2^), smoking status (never, previous, and current), alcohol consumption (never, previous, and current), physical activity (low, moderate, and high), healthy diet (yes and no), and covariates adjusted in Model 1.

^$^ Metabolic comorbidities include hypertension (yes and no), diabetes (yes and no), and dyslipidemia (yes and no).

^*^ MASLD was defined as a proton density fat fraction of 5% or greater.

^#^ MASLD was defined as a fatty liver index of 60 or greater together with at least one cardiometabolic risk factor and low alcohol consumption.

Supplementary Table 7 Associations of biological aging with incident hospital-diagnosed MASLD: Exclude MASLD cases diagnosed within the first 5 years of follow-up (N = 246,560)**.**

| **Biological aging** | **Cases/participants** | **Model 1** | |  | **Model 2** | |  | **Model 2 + metabolic comorbidities^*^** | |
| --- | --- | --- | --- | --- | --- | --- | --- | --- | --- |
|  |  | **HR (95% CI)** | ***P* value** |  | **HR (95% CI)** | ***P* value** |  | **HR (95% CI)** | ***P* value** |
| PhenoAge acceleration (continuous) | 2,805/246,560 | 1.06 (1.05, 1.06) | <0.001 |  | 1.03 (1.02, 1.04) | <0.001 |  | 1.02 (1.02, 1.03) | <0.001 |
| **PhenoAge acceleration (Tertiles)** |  |  |  |  |  |  |  |  |  |
| T1 | 515/81,450 | 1.00 (ref) |  |  | 1.00 (ref) |  |  | 1.00 (ref) |  |
| T2 | 794/81,386 | 1.50 (1.34, 1.68) | <0.001 |  | 1.22 (1.09, 1.37) | <0.001 |  | 1.22 (1.09, 1.37) | <0.001 |
| T3 | 1,496/83,724 | 2.59 (2.34, 2.87) | <0.001 |  | 1.60 (1.44, 1.78) | <0.001 |  | 1.54 (1.39, 1.72) | <0.001 |
| Non-accelerated aging | 1,011/137,538 | 1.00 (ref) |  |  | 1.00 (ref) |  |  | 1.00 (ref) |  |
| Accelerated aging | 1,794/109,022 | 2.09 (1.93, 2.26) | <0.001 |  | 1.45 (1.33, 1.57) | <0.001 |  | 1.41 (1.29, 1.53) | <0.001 |
| KDMAge acceleration (continuous) | 2,805/246,560 | 1.08 (1.07, 1.09) | <0.001 |  | 1.03 (1.02, 1.04) | <0.001 |  | 1.02 (1.01, 1.04) | <0.001 |
| **KDMAge acceleration (Tertiles)** |  |  |  |  |  |  |  |  |  |
| T1 | 638/81,419 | 1.00 (ref) |  |  | 1.00 (ref) |  |  | 1.00 (ref) |  |
| T2 | 968/81,350 | 1.42 (1.27, 1.58) | <0.001 |  | 1.12 (1.00, 1.25) | 0.050 |  | 1.10 (0.98, 1.23) | 0.101 |
| T3 | 1,199/83,791 | 2.30 (1.99, 2.67) | <0.001 |  | 1.47 (1.26, 1.70) | <0.001 |  | 1.40 (1.20, 1.63) | <0.001 |
| Non-accelerated aging | 1,623/164,139 | 1.00 (ref) |  |  | 1.00 (ref) |  |  | 1.00 (ref) |  |
| Accelerated aging | 1,182/82,421 | 1.90 (1.67, 2.18) | <0.001 |  | 1.36 (1.19, 1.56) | <0.001 |  | 1.30 (1.13, 1.49) | <0.001 |

Abbreviation: CI, confidence interval; HR, hazard ratio; KDM, Klemera-Doubal method; MASLD, metabolic dysfunction-associated steatotic liver disease.

Model 1: adjusted for age (continuous, year), sex (male and female), race and ethnicity (White ethnicity or race and Others), education (less than high school, high school or equivalent, and college and above), and Townsend deprivation index (continuous);

Model 2: Adjusted for body mass index (continuous, kg/m^2^), smoking status (never, previous, and current), alcohol consumption (never, previous, and current), physical activity (low, moderate, and high), healthy diet (yes and no), and covariates adjusted in Model 1.

^*^ Metabolic comorbidities include hypertension (yes and no), diabetes (yes and no), and dyslipidemia (yes and no).

Supplementary Table 8 Associations of biological aging with incident hospital-diagnosed MASLD: Exclude excessive drinkers **(N = 147,701).**

| **Biological aging** | **Cases/participants** | **Model 1** | |  | **Model 2** | |  | **Model 2 + metabolic comorbidities^*^** | |
| --- | --- | --- | --- | --- | --- | --- | --- | --- | --- |
|  |  | **HR (95% CI)** | ***P* value** |  | **HR (95% CI)** | ***P* value** |  | **HR (95% CI)** | ***P* value** |
| PhenoAge acceleration (continuous) | 2,023/147,701 | 1.06 (1.05, 1.06) | <0.001 |  | 1.03 (1.02, 1.04) | <0.001 |  | 1.02 (1.01, 1.03) | <0.001 |
| **PhenoAge acceleration (Tertiles)** |  |  |  |  |  |  |  |  |  |
| T1 | 357/48,952 | 1.00 (ref) |  |  | 1.00 (ref) |  |  | 1.00 (ref) |  |
| T2 | 559/47,873 | 1.56 (1.37, 1.79) | <0.001 |  | 1.25 (1.1, 1.43) | 0.001 |  | 1.25 (1.10, 1.43) | 0.001 |
| T3 | 1,107/50,876 | 2.75 (2.44, 3.11) | <0.001 |  | 1.58 (1.39, 1.8) | <0.001 |  | 1.50 (1.32, 1.71) | <0.001 |
| Non-accelerated aging | 703/82,040 | 1.00 (ref) |  |  | 1.00 (ref) |  |  | 1.00 (ref) |  |
| Accelerated aging | 1,320/65,661 | 2.20 (2.00, 2.41) | <0.001 |  | 1.43 (1.30, 1.58) | <0.001 |  | 1.37 (1.24, 1.52) | <0.001 |
| KDMAge acceleration (continuous) | 3,254/147,701 | 1.08 (1.07, 1.09) | <0.001 |  | 1.03 (1.01, 1.04) | <0.001 |  | 1.02 (1.00, 1.03) | 0.011 |
| **KDMAge acceleration (Tertiles)** |  |  |  |  |  |  |  |  |  |
| T1 | 501/58,805 | 1.00 (ref) |  |  | 1.00 (ref) |  |  | 1.00 (ref) |  |
| T2 | 860/55,053 | 1.62 (1.43, 1.83) | <0.001 |  | 1.23 (1.08, 1.39) | 0.001 |  | 1.21 (1.06, 1.38) | 0.005 |
| T3 | 662/39,343 | 2.37 (1.95, 2.86) | <0.001 |  | 1.43 (1.18, 1.73) | <0.001 |  | 1.35 (1.11, 1.64) | 0.003 |
| Non-accelerated aging | 1368/109,039 | 1.00 (ref) |  |  | 1.00 (ref) |  |  | 1.00 (ref) |  |
| Accelerated aging | 655/38,662 | 1.89 (1.58, 2.25) | <0.001 |  | 1.30 (1.08, 1.55) | 0.004 |  | 1.22 (1.02, 1.46) | 0.03 |

Abbreviation: CI, confidence interval; HR, hazard ratio; KDM, Klemera-Doubal method; MASLD, metabolic dysfunction-associated steatotic liver disease.

Model 1: adjusted for age (continuous, year), sex (male and female), race and ethnicity (White ethnicity or race and Others), education (less than high school, high school or equivalent, and college and above), and Townsend deprivation index (continuous);

Model 2: Adjusted for body mass index (continuous, kg/m^2^), smoking status (never, previous, and current), alcohol consumption (never, previous, and current), physical activity (low, moderate, and high), healthy diet (yes and no), and covariates adjusted in Model 1.

^*^ Metabolic comorbidities include hypertension (yes and no), diabetes (yes and no), and dyslipidemia (yes and no).

Supplementary Table 9 Summary of missing data of covariates for multiple imputation

| **Covariates** | **Number** |
| --- | --- |
| Race and ethnicity | 1,473 |
| Education | 3,640 |
| Smoking status | 1,515 |
| Alcohol consumption | 708 |
| Physical activity | 71,933 |
| Body mass index | 673 |
| Townsend deprivation index | 378 |

Supplementary Table 10 Associations of biological aging with incident hospital-diagnosed MASLD using the imputed dataset **(N = 323,009)**

| **Biological aging** | **Cases/participants** | **Model 1** | |  | **Model 2** | |  | **Model 2 + metabolic comorbidities^*^** | |
| --- | --- | --- | --- | --- | --- | --- | --- | --- | --- |
|  |  | **HR (95% CI)** | ***P* value** |  | **HR (95% CI)** | ***P* value** |  | **HR (95% CI)** | ***P* value** |
| PhenoAge acceleration (continuous) | 4,597/323,009 | 1.06 (1.05, 1.06) | <0.001 |  | 1.03 (1.03, 1.04) | <0.001 |  | 1.03 (1.02, 1.03) | <0.001 |
| **PhenoAge acceleration (Tertiles)** |  |  |  |  |  |  |  |  |  |
| T1 | 797/106,593 | 1.00 (ref) |  |  | 1.00 (ref) |  |  | 1.00 (ref) |  |
| T2 | 1,331/106,593 | 1.61 (1.48, 1.76) | <0.001 |  | 1.31 (1.20, 1.43) | <0.001 |  | 1.31 (1.20, 1.43) | <0.001 |
| T3 | 2,469/109,823 | 2.71 (2.50, 2.94) | <0.001 |  | 1.67 (1.53, 1.82) | <0.001 |  | 1.60 (1.47, 1.75) | <0.001 |
| Non-accelerated aging | 1,582/176,410 | 1.00 (ref) |  |  | 1.00 (ref) |  |  | 1.00 (ref) |  |
| Accelerated aging | 3,015/146,599 | 2.11 (1.98, 2.25) | <0.001 |  | 1.46 (1.37, 1.56) | <0.001 |  | 1.41 (1.32, 1.51) | <0.001 |
| KDMAge acceleration (continuous) | 4,597/323,009 | 1.08 (1.07, 1.09) | <0.001 |  | 1.03 (1.02, 1.04) | <0.001 |  | 1.02 (1.01, 1.03) | 0.011 |
| **KDMAge acceleration (Tertiles)** |  |  |  |  |  |  |  |  |  |
| T1 | 1,040/106,593 | 1.00 (ref) |  |  | 1.00 (ref) |  |  | 1.00 (ref) |  |
| T2 | 1,615/106,593 | 1.44 (1.33, 1.57) | <0.001 |  | 1.14 (1.04, 1.24) | 0.004 |  | 1.10 (1.01, 1.20) | 0.036 |
| T3 | 1,942/109,823 | 2.29 (2.03, 2.59) | <0.001 |  | 1.48 (1.31, 1.66) | <0.001 |  | 1.37 (1.21, 1.55) | <0.001 |
| Non-accelerated aging | 2,745/218,787 | 1.00 (ref) |  |  | 1.00 (ref) |  |  | 1.00 (ref) |  |
| Accelerated aging | 1,852/104,222 | 1.83 (1.64, 2.03) | <0.001 |  | 1.31 (1.17, 1.46) | <0.001 |  | 1.23 (1.10, 1.37) | <0.001 |

Abbreviation: CI, confidence interval; HR, hazard ratio; KDM, Klemera-Doubal method; MASLD, metabolic dysfunction-associated steatotic liver disease.

Model 1: adjusted for age (continuous, year), sex (male and female), race and ethnicity (White ethnicity or race and Others), education (less than high school, high school or equivalent, and college and above), and Townsend deprivation index (continuous);

Model 2: Adjusted for body mass index (continuous, kg/m^2^), smoking status (never, previous, and current), alcohol consumption (never, previous, and current), physical activity (low, moderate, and high), healthy diet (yes and no), and covariates adjusted in Model 1.

^*^ Metabolic comorbidities include hypertension (yes and no), diabetes (yes and no), and dyslipidemia (yes and no).

Supplementary Table 11 Cardiometabolic biomarkers at baseline survey **(N = 242,834)**

| **Biomarkers** | **Total  (N = 242,834)** | **No MASLD  (N = 239,665)** | **Incident hospital-diagnosed MASLD  (N = 3,169)** |
| --- | --- | --- | --- |
| **Glucose homeostasis** |  |  |  |
| Glucose, mean (SD), mg/dL | 91.73 (20.79) | 91.62 (20.51) | 100.33 (35.13) |
| HbA1c, mean (SD), % | 5.42 (0.57) | 5.42 (0.57) | 5.72 (0.89) |
| TyG index | 4.22 (0.56) | 4.21 (0.56) | 4.55 (0.60) |
| **Lipid profiles** |  |  |  |
| LDL-C, mean (SD), mg/dL | 3.57 (0.86) | 3.57 (0.86) | 3.46 (0.92) |
| HDL-C, mean (SD), mg/dL | 1.46 (0.38) | 1.46 (0.38) | 1.28 (0.35) |
| TC, mean (SD), mg/dL | 5.71 (1.12) | 5.71 (1.12) | 5.50 (1.23) |
| TG, mean (SD), mg/dL | 1.73 (1.01) | 1.72 (1.00) | 2.23 (1.23) |
| **Blood pressure** |  |  |  |
| SBP, mean (SD), mm Hg | 137.23 (18.36) | 137.19 (18.37) | 140.46 (18.01) |
| DBP, mean (SD), mm Hg | 82.12 (10.06) | 82.09 (10.06) | 84.59 (10.08) |
| **Inflammation** |  |  |  |
| CRP, mean (SD), mg/dL | 0.24 (0.39) | 0.23 (0.39) | 0.38 (0.49) |
| Urate, mean (SD), μmol/L | 15.08 (3.75) | 15.08 (3.74) | 15.20 (4.07) |
| GGT, mean (SD), U/L | 36.20 (38.48) | 35.76 (37.14) | 69.85 (89.39) |
| **Liver function** |  |  |  |
| AST, mean (SD), U/L | 26.06 (9.57) | 25.97 (9.37) | 32.90 (17.96) |
| ALT, mean (SD), U/L | 23.34 (13.66) | 23.18 (13.36) | 35.61 (25.27) |

Abbreviation: ALT, alanine aminotransferase; AST, aspartate aminotransferase; BMI, body mass index; CRP, C-reactive protein; DBP, diastolic blood pressure; GGT, gamma-glutamyltransferase; HbA1c, glycosylated hemoglobin; HDL-C, high-density lipoprotein cholesterol; LDL-C, low-density lipoprotein cholesterol; MASLD, metabolic dysfunction-associated steatotic liver disease; SD, standard deviation; TC, total cholesterol; TG, triglyceride; TyG, triglyceride-glucose.

Supplementary Table 12 Associations between biological aging and cardiometabolic biomarkers **(N = 242,834)**

| **Biomarkers** | **PhenoAge acceleration** | |  | **KDMAge acceleration** | |
| --- | --- | --- | --- | --- | --- |
|  | ***β* (95% CI)** | ***P* value** |  | ***β* (95% CI)** | ***P* value** |
| **Glucose homeostasis** |  |  |  |  |  |
| Glucose | 4.43 (4.29, 4.58) | <0.001 |  | 3.04 (2.78, 3.30) | <0.001 |
| HbA1c | 0.16 (0.15, 0.16) | <0.001 |  | 0.15 (0.14, 0.15) | <0.001 |
| TyG index | 0.06 (0.05, 0.06) | <0.001 |  | 0.19 (0.18, 0.19) | <0.001 |
| **Lipid profiles** |  |  |  |  |  |
| LDL-C | -0.07 (-0.08, -0.06) | <0.001 |  | 0.30 (0.29, 0.31) | <0.001 |
| HDL-C | -0.01 (-0.01, -0.01) | <0.001 |  | -0.01 (-0.01, -0.01) | <0.001 |
| TC | -0.09 (-0.10, -0.08) | <0.001 |  | 0.41 (0.40, 0.43) | <0.001 |
| TG | 0.002 (-0.006, 0.010) | 0.561 |  | 0.32 (0.31, 0.34) | <0.001 |
| **Blood pressure** |  |  |  |  |  |
| SBP | 0.58 (0.43, 0.72) | <0.001 |  | 12.56 (12.35, 12.77) | <0.001 |
| DBP | 0.19 (0.10, 0.27) | <0.001 |  | 5.61 (5.49, 5.73) | <0.001 |
| **Inflammation** |  |  |  |  |  |
| CRP | 0.24 (0.24, 0.25) | <0.001 |  | 0.19 (0.18, 0.19) | <0.001 |
| Urate | 0.54 (0.51, 0.57) | <0.001 |  | 1.46 (1.41, 1.50) | <0.001 |
| GGT | 5.14 (4.83, 5.46) | <0.001 |  | 10.88 (10.41, 11.35) | <0.001 |
| **Liver function** |  |  |  |  |  |
| AST | 0.56 (0.48, 0.64) | <0.001 |  | 1.70 (1.59, 1.82) | <0.001 |
| ALT | 0.16 (0.05, 0.27) | 0.004 |  | 3.27 (3.11, 3.43) | <0.001 |

Abbreviation: CI, confidence interval; CRP, C-reactive protein; DBP, diastolic blood pressure; GGT, gamma-glutamyltransferase; HbA1c, glycosylated hemoglobin; HDL-C, high-density lipoprotein cholesterol; LDL-C, low-density lipoprotein cholesterol; TC, total cholesterol; TG, triglyceride; TyG, triglyceride-glucose.

Models were adjusted for age (continuous, year), sex (male and female), race (White ethnicity or race and Others), education (less than high school, high school or equivalent, and college and above), Townsend deprivation index (continuous), body mass index (continuous, kg/m^2^), smoking status (never, previous, and current), alcohol consumption (never, previous, and current), physical activity (low, moderate, and high), and healthy diet (yes and no).

Supplementary Table 13 Associations of lifestyles with incident hospital-diagnosed MASLD during follow-up **(N = 258,557)**

| **Behaviors** | **Model 1** | |  | **Model 2** | |  | **Model 2 + metabolic comorbidities^*^** | |
| --- | --- | --- | --- | --- | --- | --- | --- | --- |
|  | **HR (95% CI)** | ***P* value** |  | **HR (95% CI)** | ***P* value** |  | **HR (95% CI)** | ***P* value** |
| **Current smoking** | 1.29 (1.17, 1.43) | <0.001 |  | 1.44 (1.30, 1.59) | <0.001 |  | 1.43 (1.29, 1.58) | <0.001 |
| **Drinking** | 1.23 (1.04, 1.44) | 0.015 |  | 1.18 (1.01, 1.40) | 0.043 |  | 1.15 (0.98, 1.36) | 0.088 |
| **Poor diet** | 1.31 (1.18, 1.46) | <0.001 |  | 1.17 (1.05, 1.31) | 0.004 |  | 1.17 (1.05, 1.30) | 0.004 |
| **Low physical activity** | 1.44 (1.33, 1.56) | <0.001 |  | 1.09 (1.00, 1.18) | 0.038 |  | 1.06 (0.98, 1.16) | 0.136 |

Abbreviations: CI, confidence interval; HR, hazard ratio; MASLD, metabolic dysfunction-associated steatotic liver disease.

Model 1: adjusted for age (continuous, year), sex (male and female), race and ethnicity (White ethnicity or race and Others), education (less than high school, high school or equivalent, and college and above), and Townsend deprivation index (continuous);

Model 2: Adjusted for body mass index (continuous, kg/m^2^), smoking status (never, previous, and current), alcohol consumption (never, previous, and current), physical activity (low, moderate, and high), healthy diet (yes and no), and covariates adjusted in Model 1.

^*^ Metabolic comorbidities include hypertension (yes and no), diabetes (yes and no), and dyslipidemia (yes and no).

Supplementary Table 14 Associations of lifestyles with biological age acceleration **(N = 258,557)**

| **Behaviors** | **Model 1** | |  | **Model 2** | |  | **Model 2 + metabolic comorbidities^*^** | |
| --- | --- | --- | --- | --- | --- | --- | --- | --- |
|  | **HR (95% CI)** | ***P* value** |  | **HR (95% CI)** | ***P* value** |  | **HR (95% CI)** | ***P* value** |
| **PhenoAge acceleration** |  |  |  |  |  |  |  |  |
| Current smoking | 2.22 (2.16, 2.29) | <0.001 |  | 2.53 (2.46, 2.61) | <0.001 |  | 2.55 (2.47, 2.62) | <0.001 |
| Drinking | 1.20 (1.15, 1.26) | <0.001 |  | 1.22 (1.16, 1.28) | <0.001 |  | 1.19 (1.14, 1.25) | <0.001 |
| Poor diet | 1.36 (1.32, 1.39) | <0.001 |  | 1.21 (1.18, 1.24) | <0.001 |  | 1.21 (1.18, 1.23) | <0.001 |
| Low physical activity | 1.42 (1.39, 1.45) | <0.001 |  | 1.17 (1.14, 1.20) | <0.001 |  | 1.16 (1.14, 1.19) | <0.001 |
| **KDMAge acceleration** |  |  |  |  |  |  |  |  |
| Current smoking | 1.39 (1.33, 1.45) | <0.001 |  | 1.60 (1.53, 1.67) | <0.001 |  | 1.66 (1.58, 1.74) | <0.001 |
| Drinking | 1.00 (0.92, 1.09) | 0.979 |  | 1.07 (0.98, 1.18) | 0.121 |  | 1.16 (1.06, 1.28) | 0.001 |
| Poor diet | 1.53 (1.47, 1.58) | <0.001 |  | 1.34 (1.29, 1.38) | <0.001 |  | 1.32 (1.28, 1.38) | <0.001 |
| Low physical activity | 1.28 (1.24, 1.33) | <0.001 |  | 1.05 (1.01, 1.08) | 0.013 |  | 1.06 (1.02, 1.10) | 0.003 |

Abbreviations: CI, confidence interval; HR, hazard ratio; MASLD, metabolic dysfunction-associated steatotic liver disease.

Model 1: adjusted for age (continuous, year), sex (male and female), race and ethnicity (White ethnicity or race and Others), education (less than high school, high school or equivalent, and college and above), and Townsend deprivation index (continuous);

Model 2: Adjusted for body mass index (continuous, kg/m^2^), smoking status (never, previous, and current), alcohol consumption (never, previous, and current), physical activity (low, moderate, and high), healthy diet (yes and no), and covariates adjusted in Model 1.

^*^ Metabolic comorbidities include hypertension (yes and no), diabetes (yes and no), and dyslipidemia (yes and no).


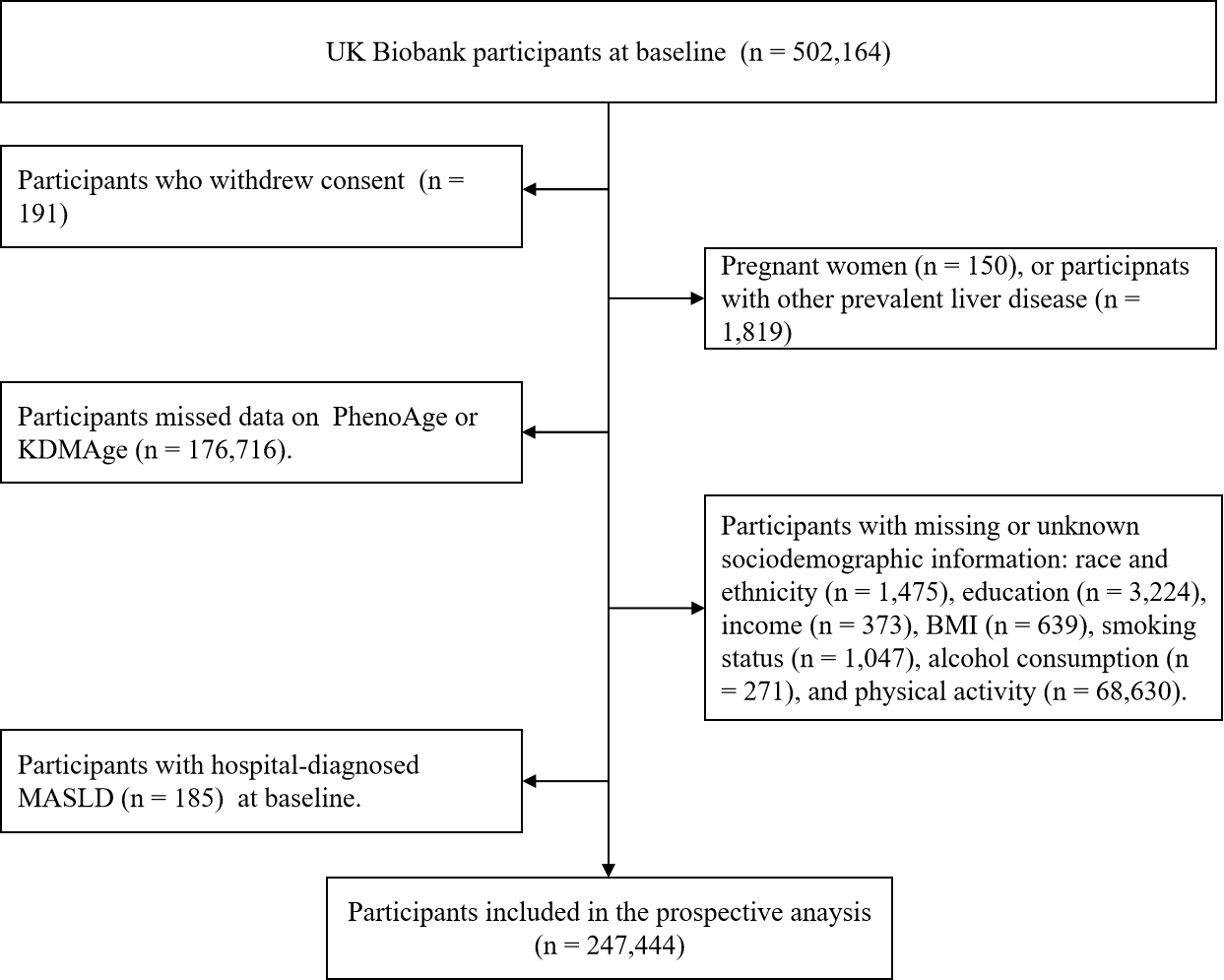


Supplementary Figure 1 Flowchart of selection of participants in the UK Biobank. Abbreviation: BMI, body mass index; MASLD, metabolic dysfunction-associated steatotic liver disease.


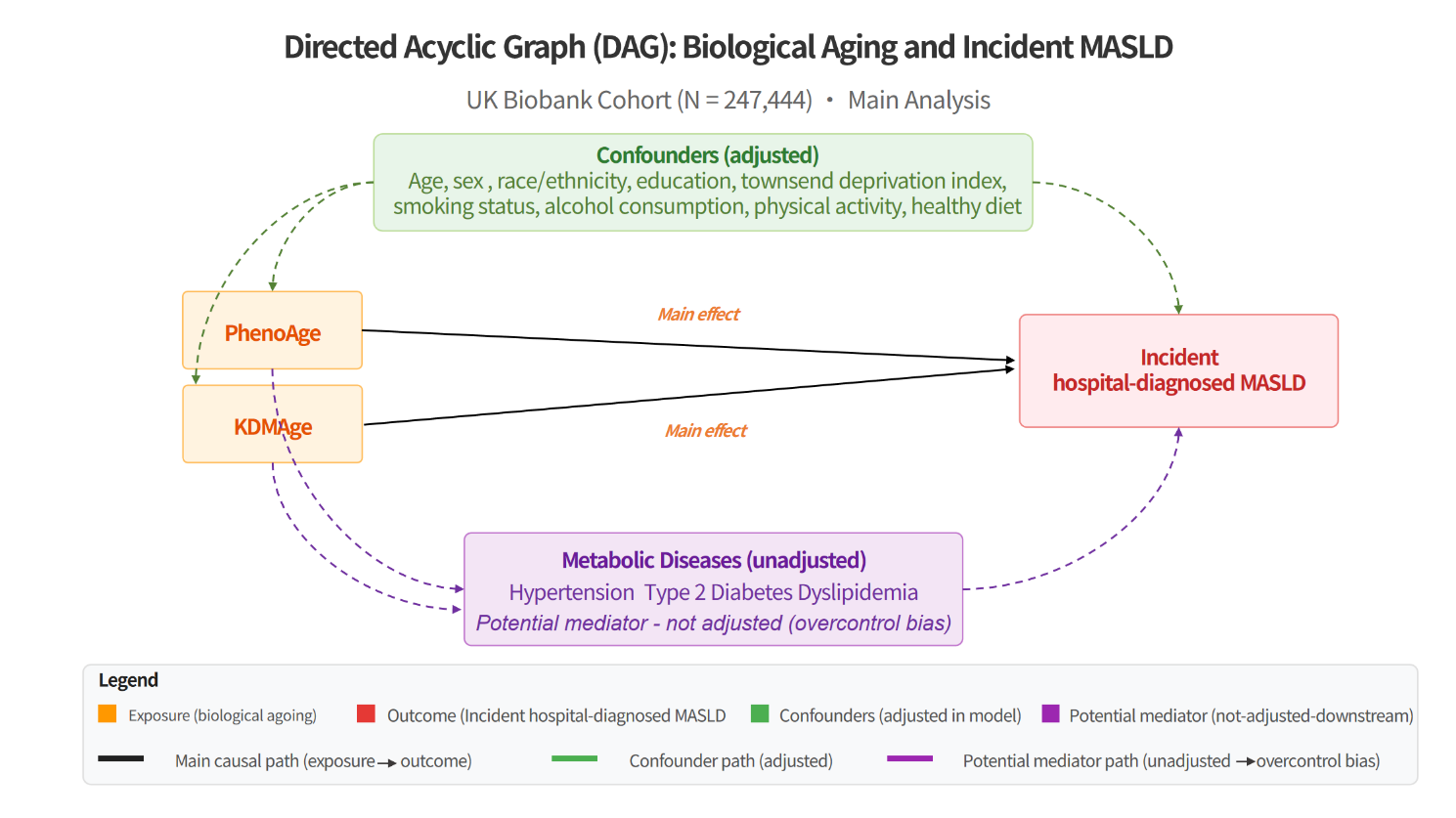
Supplementary Figure 2 Directed acyclic graph illustrating the hypothesized causal relationships between biological aging and incident MASLD. Abbreviation: BMI, body mass index; MASLD, metabolic dysfunction-associated steatotic liver disease.


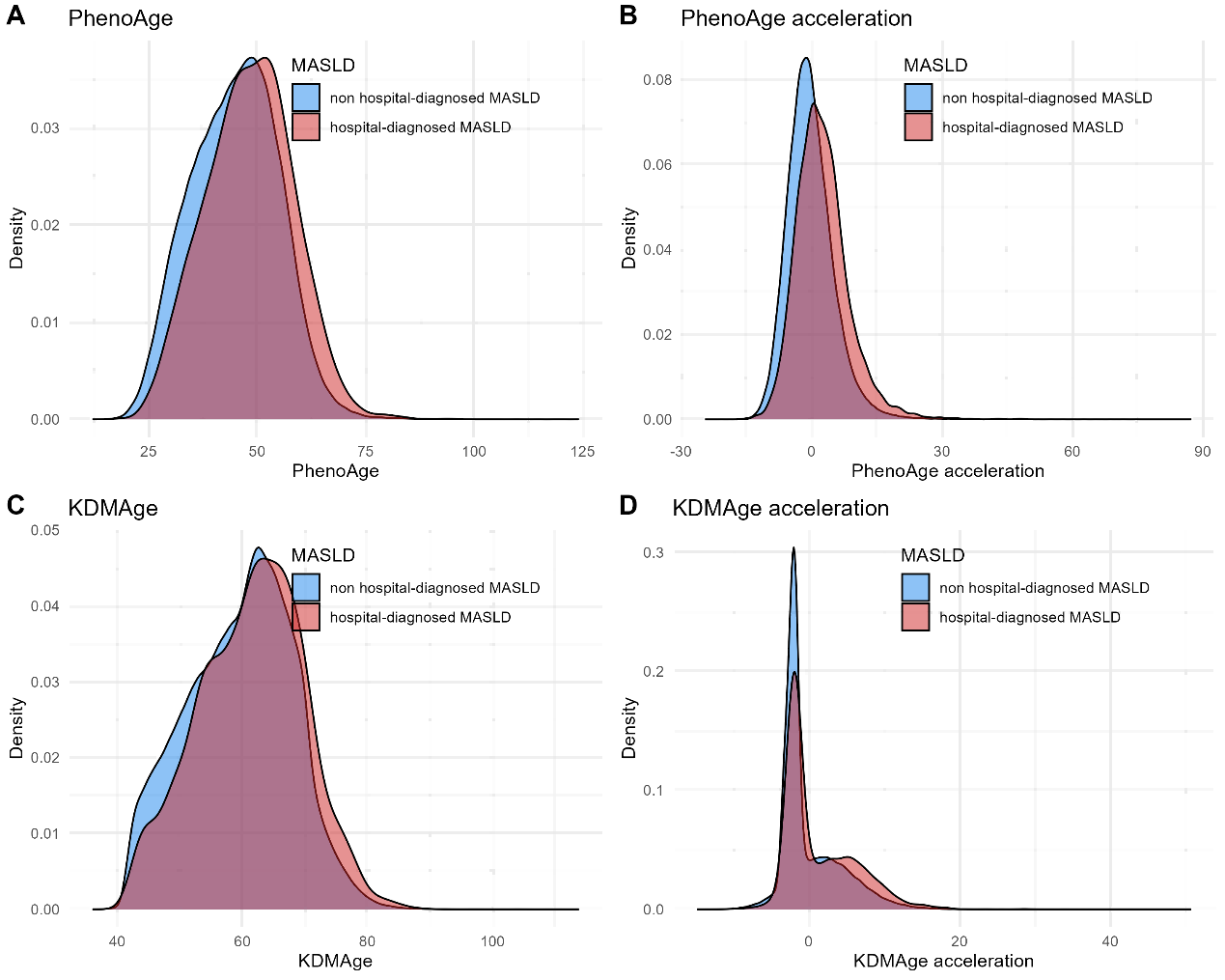


Supplementary Figure 3 The distribution of biological age and age acceleration across non-MASLD and hospital-diagnosed MASLD. Abbreviation: MASLD, metabolic dysfunction-associated steatotic liver disease. A) The distribution of PhenoAge in UK Biobank; B) The distribution of PhenoAge acceleration in UK Biobank; C) The distribution of KDMAge in UK Biobank; D) The distribution of KDMAge acceleration in UK Biobank.


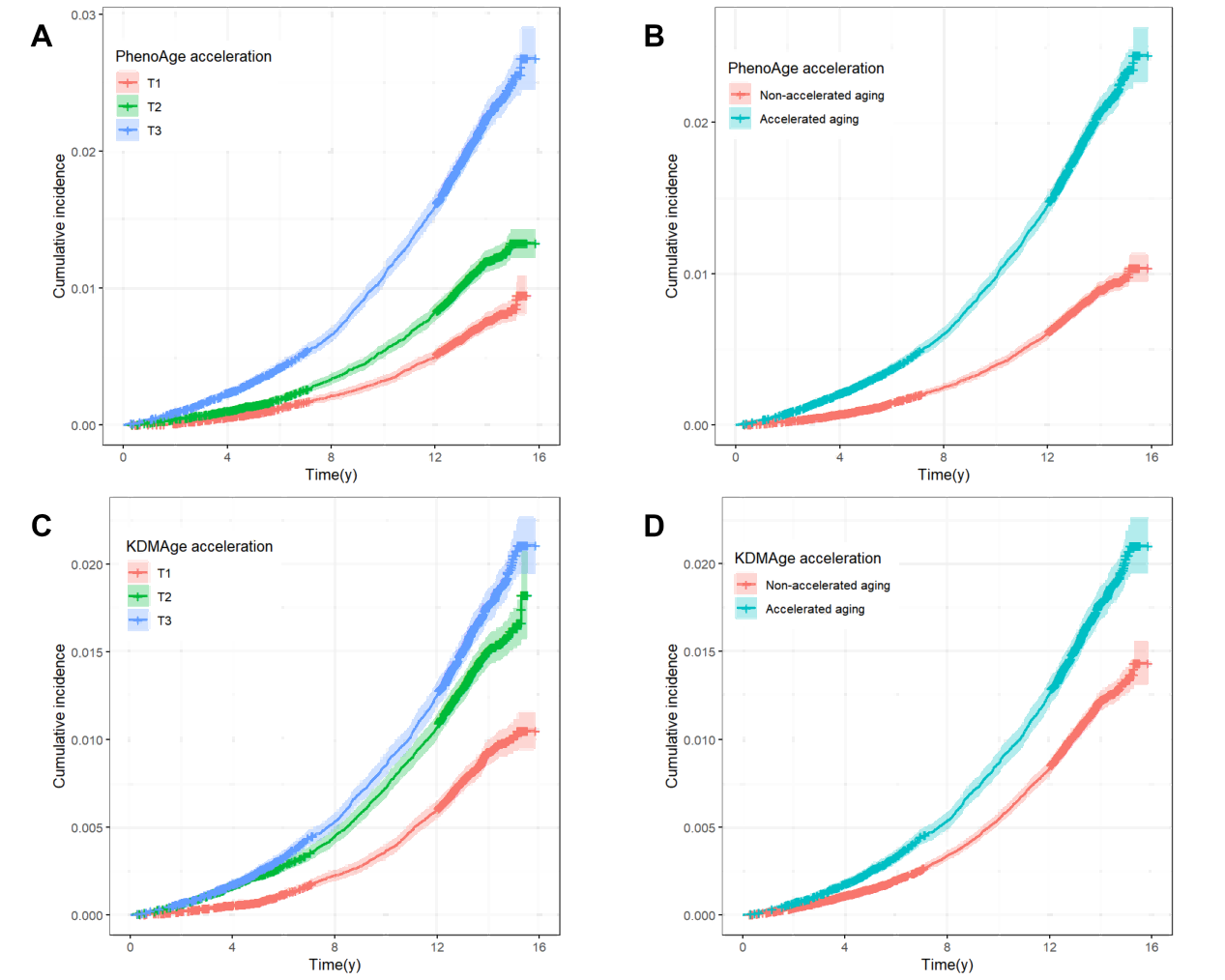


Supplementary Figure 4 Kaplan-Meier survival estimates according to biological age and age acceleration for the probability of incident hospital-diagnosed MASLD in UK Biobank. Abbreviation: MASLD, metabolic dysfunction-associated steatotic liver disease


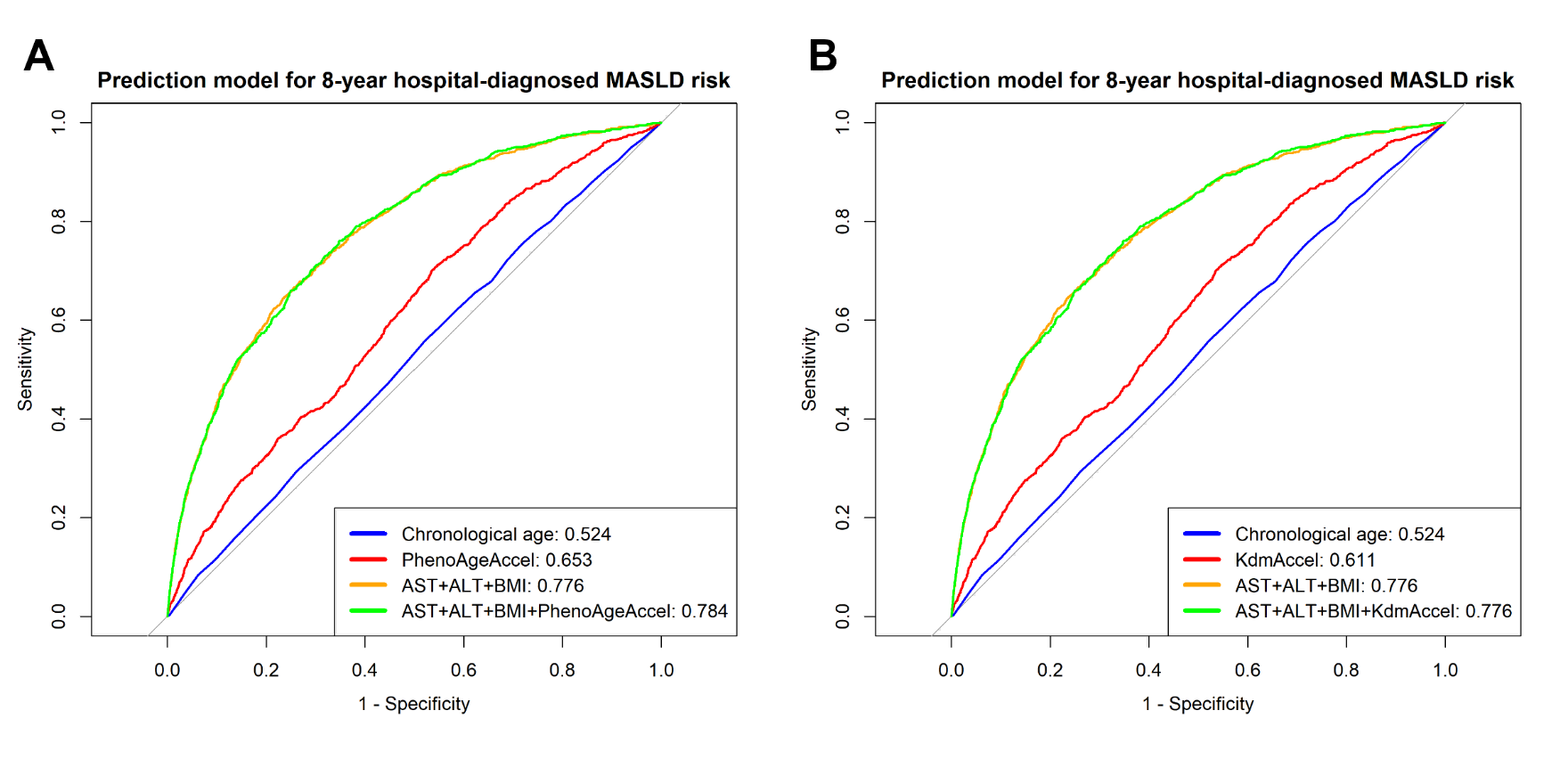


Supplementary Figure 5 Receiver-operating characteristic curves for 8-year hospital-diagnosed MASLD risk**.** A) PhenoAge acceleration alone showed better prediction ability than chronological age (AUROC: 0.653 vs 0.524; *P* < 0.001). When separately adding PhenoAge acceleration into the base model, PhenoAge acceleration yielded an increase in the AUROC (AUROC: 0.784 vs 0.776; *P* = 0.003), despite the small difference; B) KDMAge acceleration alone showed better prediction ability than chronological age (AUROC: 0.611 vs 0.524; *P* < 0.001). When separately adding KDMAge acceleration into the base model, no significant increase in AUROC was observed (*P* = 0.682). Abbreviation: ALT, alanine aminotransferase; AST, aspartate aminotransferase; AUROC, area under the receiver operating characteristic curve; BMI, body mass index; MASLD, metabolic dysfunction-associated steatotic liver disease.
